# Cerebrospinal fluid penetration of cycloserine/terizidone and clofazimine in patients with pulmonary TB

**DOI:** 10.1128/aac.00931-25

**Published:** 2025-10-21

**Authors:** Caryn M. Upton, Jose M. Calderin, Andreas H. Diacon, Martin J. Boeree, Paolo Denti, Lubbe Wiesner, Tracy Kellermann, Megan McCulloch, Rob Aarnoutse

**Affiliations:** 1 Radboud University Medical Center, Radboud Institute for Medical Innovation 6034, Nijmegen, the Netherlands; 2 TASK 728678 , Cape Town, South Africa; 3 Division of Clinical Pharmacology, Department of Medicine, University of Cape Town, Cape Town, South Africa; 4 Division of Clinical Pharmacology, Department of Medicine, Stellenbosch University 26697, Stellenbosch, South Africa; City St George's, University of London, London, United Kingdom

**Keywords:** tuberculous meningitis, pharmacokinetics, cerebrospinal fluid, terizidone, cycloserine, clofazimine, NONMEM, population pharmacokinetics, population modeling

## Abstract

Tuberculous meningitis (TBM) treatment outcomes are poor, partly due to suboptimal drug penetration into the cerebrospinal fluid (CSF). Little is known about the CSF pharmacokinetics of many TB drugs, both established and new. This study investigated the CSF penetration of cycloserine (administered as terizidone) and clofazimine, two core second-line drugs for drug-resistant tuberculosis (TB). We recruited participants with pulmonary drug-resistant TB, but without TBM, receiving terizidone and/or clofazimine for at least 2 weeks and collected serial plasma samples and a single CSF sample. Drug concentrations were quantified with validated liquid chromatography-tandem mass spectrometry methods. Pharmacokinetic parameters were determined using noncompartmental analysis, and population pharmacokinetic modeling was used to estimate the partition coefficient and equilibration half-life. Data were available from 27 participants, with a median age of 36 (range 20–60) and a weight of 52 kg (30–73 kg), who contributed 216 plasma and 27 CSF samples. The plasma pharmacokinetics of both drugs was in line with previous reports. Terizidone, measured as cycloserine, achieved CSF exposure of 69% relative to plasma, with plasma and CSF concentrations equilibrating with a half-life of 4.7 hours. Clofazimine CSF penetration was 0.13% of plasma exposure, with an equilibration half-life of 55.4 hours. Cycloserine and clofazimine concentrations in CSF approximated their estimated unbound (active) concentration in plasma, thus suggesting good penetration of the unbound drug into the CSF, supporting their potential use in TBM regimens. This study demonstrates a feasible and reproducible method for effective assessment of CSF drug penetration for CNS infections.

## INTRODUCTION

Tuberculous meningitis (TBM) is the most lethal form of TB and affects 4.6% of patients diagnosed with TB annually (1). There is a high mortality rate of 25% globally, increasing to 65% with severe disease and to 53% in patients with HIV co-infection (2). In patients with TBM resistant to one or more first-line drugs, outcomes are worse (3–5). For drug-susceptible TBM in adults, the World Health Organization (WHO) guidelines endorse a four-drug regimen of isoniazid, rifampicin, pyrazinamide, and ethambutol (HRZE) for 2 months, followed by 4–12 months of isoniazid and rifampicin at doses recommended for pulmonary tuberculosis (4, 6). This guidance has not been updated since 2010 (7). Several first-line agents used in pulmonary TB lack sufficient concentrations in the central nervous system (CNS), which likely contributes to the persistently poor outcomes seen in TBM (8). Without adequate CNS and cerebrospinal fluid (CSF) penetration, even potent TB drugs may fail in treating TBM. Data on the penetration of uncharacterized TB drugs could support treatment strategies that improve outcomes.

Recruitment of patients with TBM into experimental clinical trials is challenging due to diagnostic uncertainty, the need for immediate treatment, difficulties in obtaining informed consent, and high mortality, limiting follow-up and long-term monitoring (1, 9). Investigating CSF drug penetration in patients with pulmonary TB with intact blood-brain and blood-CSF barriers (BBB, BCSFB) enables characterization of CSF exposure, drug penetration, and pharmacokinetic variability in a target group without disease-related confounders. Also, an intact BBB and BCSFB may be more representative of CNS penetration later in treatment, where barrier recovery is expected to begin within 2 weeks with effective treatment (10). This exploratory approach establishes a baseline for future studies of regimens and related PK targets in TBM.

Evaluation of WHO Group A drugs bedaquiline, linezolid, and fluoroquinolones has been reported (11, 12). Clofazimine, WHO endorsed based on the endTB, BEAT-tuberculosis, and STREAM-2 regimens in pulmonary TB, and terizidone, an oral prodrug that rapidly hydrolyzes into two active cycloserine molecules, are currently classified as group B agents (4, 13). Knowledge of the CSF penetration of these drugs remains limited. This study aimed to determine the CSF concentrations of terizidone (measured as cycloserine) and clofazimine in patients treated for pulmonary TB, and the relative penetration of each drug into the CSF.

## MATERIALS AND METHODS

### Study design and participants

This observational pharmacokinetic study, conducted at TASK in Cape Town, enrolled participants with pulmonary drug-resistant TB (but not TBM) who were recruited from local clinics or Brooklyn Chest Hospital. All participants had been receiving treatment with terizidone (500 or 750 mg once daily) and/or clofazimine (100 mg daily) for at least 2 weeks prior to enrollment. The study excluded patients with contraindications to lumbar puncture or those receiving prohibited medications. In the absence of prior CSF PK data, particularly for clofazimine, it was uncertain whether a single CSF sampling time point would yield measurable concentrations in all patients. To further account for potential data loss due to technical quantification challenges and incomplete plasma PK profiles, we targeted a maximum sample size of 40 per drug (14, 15).

### Procedures

Assessment included a focused neurological examination. Medication intake for the two preceding doses was self-reported. On the PK sampling day, site staff administered all TB medications, including study drugs, via directly observed therapy. Food intake was not standardized or documented. Participants underwent intensive plasma PK sampling pre-dose and at 2, 4, 6, 8, 10, 14, and 24 hours post-dose, with blood collected into EDTA tubes. A single time-matched CSF sample was collected from each participant at 2, 4, 6, or 8 hours post-dose. Lumbar punctures were performed using an atraumatic needle under aseptic conditions. CSF sampling times were sequentially assigned to participants to ensure coverage across the expected pharmacokinetic profile of the drugs. Plasma samples were centrifuged at 1,000 *× g* for 10 minutes at 5°C within 15 minutes of collection, then aliquoted (1 mL). Plasma and CSF samples were stored at −70°C or colder within 30 minutes of collection.

CSF and plasma albumin levels were measured to calculate the CSF:serum albumin ratio, a surrogate marker of blood-CSF barrier (BCSFB) permeability that may influence drug penetration (16). Plasma and CSF drug concentrations were quantified using validated liquid chromatography-tandem mass spectrometry (LC-MS/MS) assays, measuring total (protein-bound and unbound) concentrations of clofazimine and cycloserine (following terizidone administration). Clofazimine and cycloserine were analyzed at the University of Cape Town and Stellenbosch University Pharmacology Divisions, respectively. Details on bioanalytical methods can be found in Supplement S3. A very low lower limit of quantification (LLOQ) for clofazimine was selected based on protein binding, with LLOQs of 0.00781 mg/L (7.81 ng/mL) in plasma and 0.00005 mg/L (0.05 ng/mL) in CSF. For cycloserine, the LLOQs were 0.16 mg/L (160 ng/mL) in plasma and 0.2 mg/L (200 ng/mL) in CSF. Intra-laboratory validation demonstrated high accuracy (87.8%–104.7%) and low imprecision, with relative standard deviations (RSD) below 7.3% across cycloserine plasma and CSF samples. Accuracy ranged from 97.8% to 105.5%, with RSD values below 5.7% in both plasma and CSF for clofazimine.

Safety assessments included monitoring for post-lumbar puncture complications such as headaches, hypotension, back pain, or neurological symptoms. Blood pressure and vital signs were recorded pre-lumbar puncture and at regular intervals post-procedure. Participants were followed up the next day to assess for any delayed adverse events.

### Pharmacokinetic and statistical analysis

The primary endpoint was the determination of drug concentrations in CSF. Non-compartmental analysis (NCA) estimated plasma pharmacokinetic parameters using Phoenix WinNonlin Version 8.4. Population pharmacokinetic (PopPK) modeling was performed using nonlinear mixed-effects modeling in NONMEM Version 7.5.1. Plasma concentrations were characterized first, followed by the integration of CSF data, using a sequential (non-simultaneous) approach (17). Full details on the methodology for model development are provided in the Supplemental material, and a summary is included below.

For cycloserine concentrations in plasma, a new model was developed using standard procedures for model development and inclusion of covariates (18). Terizidone doses were adjusted to cycloserine equivalents, in line with previous reports (19). For clofazimine in plasma, a previous model was adapted and fitted to the current data (20). Individual plasma pharmacokinetic parameters were fixed, and CSF concentrations were interpreted by using a model approach known as the effect compartment, that is, a theoretical compartment with no transfer of mass/drug involved, whose concentrations are driven by plasma concentration with a first-order delay. This approach can estimate the CSF-to-plasma pseudo-partition coefficient (PPC_CSF-Plasma_), which determines the ratio of concentrations between the two compartments, and equilibration half-life (t1/2_Plasma-CSF_), which quantifies the equilibration delay.

Final models were used to calculate the individual values of area under concentration-time curve from 0 to 24 hours post-dose (AUC_0−24h_) and the maximum concentration (C_max_) in plasma and CSF, with descriptive statistics summarizing pharmacokinetic variability. The final parameter estimates were used to simulate plasma and CSF concentration-time profiles for the typical individual in the cohort following standard doses of terizidone (750 mg/day) and clofazimine (100 mg/day). To interpret the penetration regarding the expected unbound concentrations, we relied on previous reports of protein binding.

## RESULTS

### Participants and safety

The study recruited 27 participants; their baseline characteristics are summarized in Table 1. Recruitment challenges during the COVID-19 pandemic influenced the decision to conclude enrollment before 40 participants were reached. This convenience sample was deemed sufficient for the study’s objectives in consultation with investigators. All participants received terizidone, where 24/27 (89%) received 750 mg daily and 3/27 (11%) received 500 mg daily. In addition, 23/27 participants (85%) also received 100 mg clofazimine daily. Treatment duration ranged from at least 2 weeks to almost 2 years. However, the date of medication initiation, treatment interruption, and adherence documentation from medical records was not complete for all participants. Participants were on complex TB treatment regimens, with an average of five anti-TB drugs (range: 4–9) administered daily. Of the 9/27 participants (33%) receiving antiretroviral therapy (ART), six were on a protease inhibitor-based regimen (lopinavir/ritonavir or atazanavir/ritonavir), and three were on a dolutegravir-based regimen. No clinically significant pharmacokinetic interactions were expected between these agents and the study drugs, terizidone and clofazimine. Participants also reported taking additional concomitant medications for various indications, including chronic diseases, pain, and management of regimen adverse effects.

**TABLE 1:** Participant demographics and characteristics shown as median (range)

Characteristic	*n* = 27 (%)
Sex	
Female	16 (59%)
Male	11 (41%)
Age in years	36 (20–60)
Body composition	
Weight (kg)	52 (30–73)
Height (m)	1.63 (1.44–1.81)
Body mass index (kg/m^2^)	19.2 (14.9–26.5)
People living with HIV	13 (48%)
CD4 count	127 (36–810)
On ART	9 (33%)
CSF:serum albumin ratio	0.005 (0.002–0.0097)^*a*^

^*a*^ No participants exceeded normal age-related thresholds.

The CSF:plasma albumin ratio was available for 22 participants with a median of 0.005 (range 0.002–0.0097). No participants exceeded age-related thresholds that may have suggested a compromised CNS barrier. Adverse events reported post-procedure were expected and mild or moderate, including headache, back pain or discomfort, nausea, abdominal pain, and transient paresthesia in the buttock. No serious adverse events were observed.

### Pharmacokinetic samples and descriptive pharmacokinetic data

For cycloserine, a total of 216 plasma and 27 CSF samples were available, and for clofazimine, 184 plasma and 23 CSF samples. All measured drug concentrations were above the LLOQ, and no data imputation was performed. In plasma, cycloserine reached a geometric mean C_max_ of 49.2 mg/L and 51.5 mg/L for the 500 mg and 750 mg doses, respectively, with a median T_max_ of 3.6 and 4.3 hours. AUC_0-24h_ was 804 and 838 h*mg/L, with elimination half-lives of 17.8 and 18.7 hours, respectively. CSF concentrations ranged from 15.4 to 35.5 mg/L after 500 mg terizidone and 3.5–45.8 mg/L after 750 mg terizidone. Clofazimine had a geometric mean C_max_ of 0.49 mg/L, with a median T_max_ of 5.0 hours in plasma. AUC_0-24h_ was 9.7 h⋅mg/L, with a prolonged elimination half-life of 69 hours. The range of CSF concentrations ranged from 0.000127 to 0.00221 mg/L.

### Population pharmacokinetic modeling

#### Cycloserine

The plasma pharmacokinetics of cycloserine were best described by a one-compartment disposition model with first-order elimination and first-order absorption after a chain of transit compartments. Allometric scaling by fat-free mass (FFM) improved the model fit (ΔOFV = −33.5, *P* < 0.01) compared to allometric scaling by total body weight (ΔOFV = −20.4, *P* < 0.01). The population estimates for clearance and volume of distribution were 0.54 L/h and 13.9 L, respectively.

The model identified a statistically significant effect of age on total clearance, which was used as a proxy for age-related variations in renal function. Including this effect as an exponential function improved the model fit (ΔOFV = −8.2, df = 1, *P* < 0.01), yielding an estimated exponent of −0.0145 1/year (95% confidence interval: −0.023 to −0.006). This indicates a decrease of ~1.4% in cycloserine clearance for each year above the median age of the cohort. Further details on the modeling of age effect on total clearance are available in the Supplemental material. No other covariates were found to significantly impact the plasma pharmacokinetics of cycloserine.

Cycloserine CSF concentrations were linked to plasma concentrations with a t1/2_Plasma-CSF_ of 4.7 hours and a PPC_CSF-Plasma_ of 0.69. This means it takes approximately 24 hours (~5 half-lives) for plasma and CSF concentrations to equilibrate, and at steady state, the CSF average exposure (as evaluated with AUC) is 69% of that in plasma. Of note, these calculations are done using total (bound plus unbound) drug concentrations, without correction for protein binding in plasma or CSF. No covariates significantly affected the CSF pharmacokinetic parameters.

**Fig 1:**
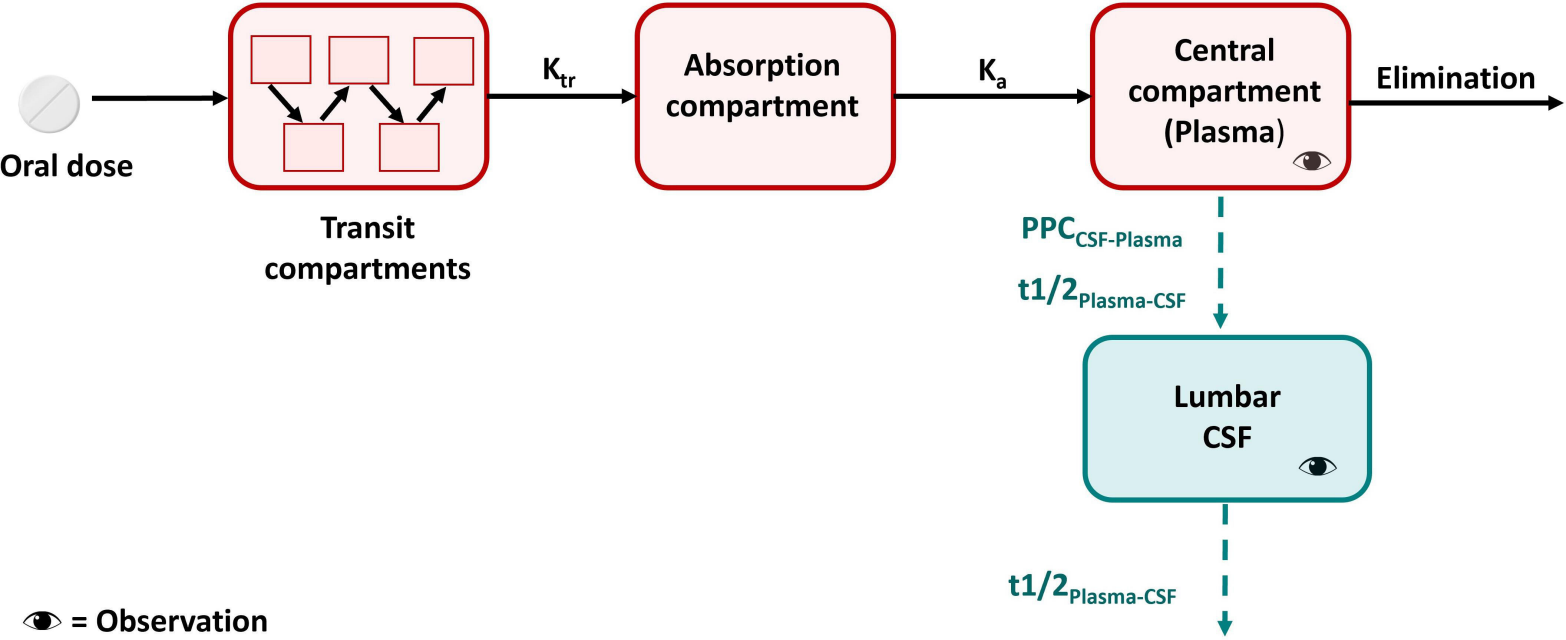
Schematic representation of the cycloserine final model. Schematic representation of the final cycloserine model. K_tr_ is the first-order rate constant for drug passage through transit compartments. t1/2_Plasma-CSF_ is the equilibration half-life between plasma and CSF, which describes how soon the change in plasma is reflected in the CSF. PPC_CSF-Plasma_ is the pseudo-partition coefficient, which represents the relative drug exposure in CSF compared to plasma at steady state.

**Fig 2:**
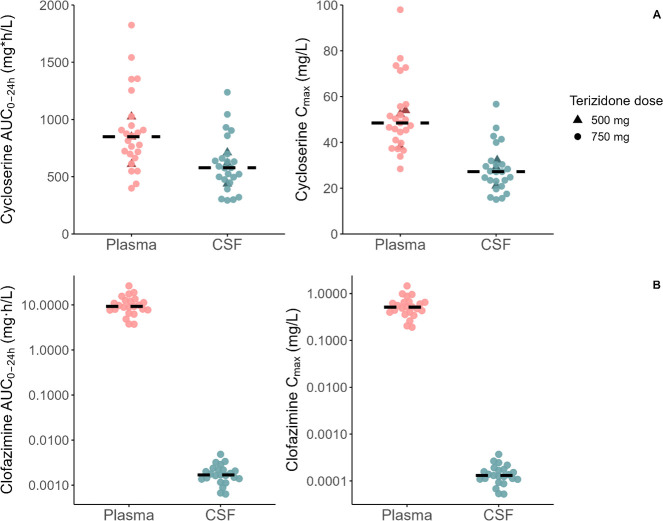
Model-derived individual cycloserine (**A**, top) and clofazimine (**B**, bottom) exposure metrics: total (protein-bound plus unbound) area under the concentration–time curve from 0 to 24 hours (AUC_0-24h_) and maximum concentration (C_max_) in plasma and CSF. The dots represent individual values, while the horizontal line indicates the median for each group.

**Fig 3:**
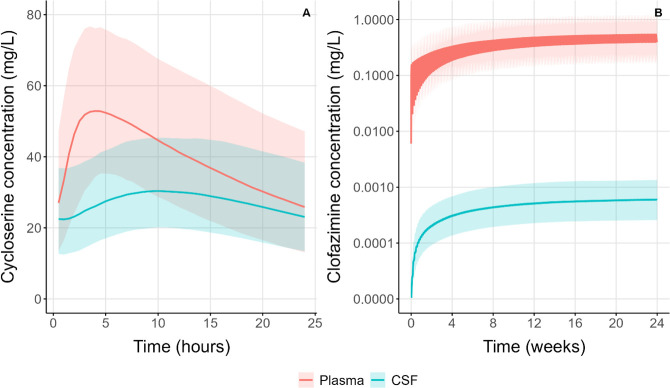
Simulated steady-state typical concentration–time profiles in plasma and CSF following (**A**) 750 mg oral daily dose of cycloserine over 24 hours (left) and (**B**) 100 mg oral daily dose of clofazimine over 24 weeks (right). Solid lines represent the median predicted concentrations for plasma and CSF, respectively; shaded areas indicate the 90% prediction intervals.

A schematic representation of the final model for cycloserine is provided in Fig. 1. The model-derived individual values of AUC_0-24h_ and C_max_ for cycloserine are depicted in Fig. 2A with a typical concentration–time profile in plasma and CSF in Fig. 3A. More results on population pharmacokinetic modeling of cycloserine can be found in the Supplemental material.

#### Clofazimine

The previous model adequately described the clofazimine observed plasma data (20). A scaling factor was included to adjust for differences in bioavailability between the cohort used to develop the original model and our study cohort. This adjustment improved model fit (ΔOFV = −9.05) and the scaling factor was estimated at 1.26 (i.e., 26% higher bioavailability than the previous study). In line with the findings from the previous model, this analysis confirmed that clofazimine accumulates in fat tissue over time on treatment (Supplemental figures).

Clofazimine CSF concentrations were linked to plasma concentrations with a t1/2_Plasma-CSF_ of 55.4 hours and a PPC_CSF-Plasma_ of 0.0013. This means it takes 277 hours (~11.5 days) for plasma and CSF concentrations to equilibrate (~5 half-lives), and at steady state, CSF exposure is 0.13% of plasma exposure, without correction for protein binding in plasma. No covariates significantly affected the CSF pharmacokinetic parameters for clofazimine.

**Fig 4:**
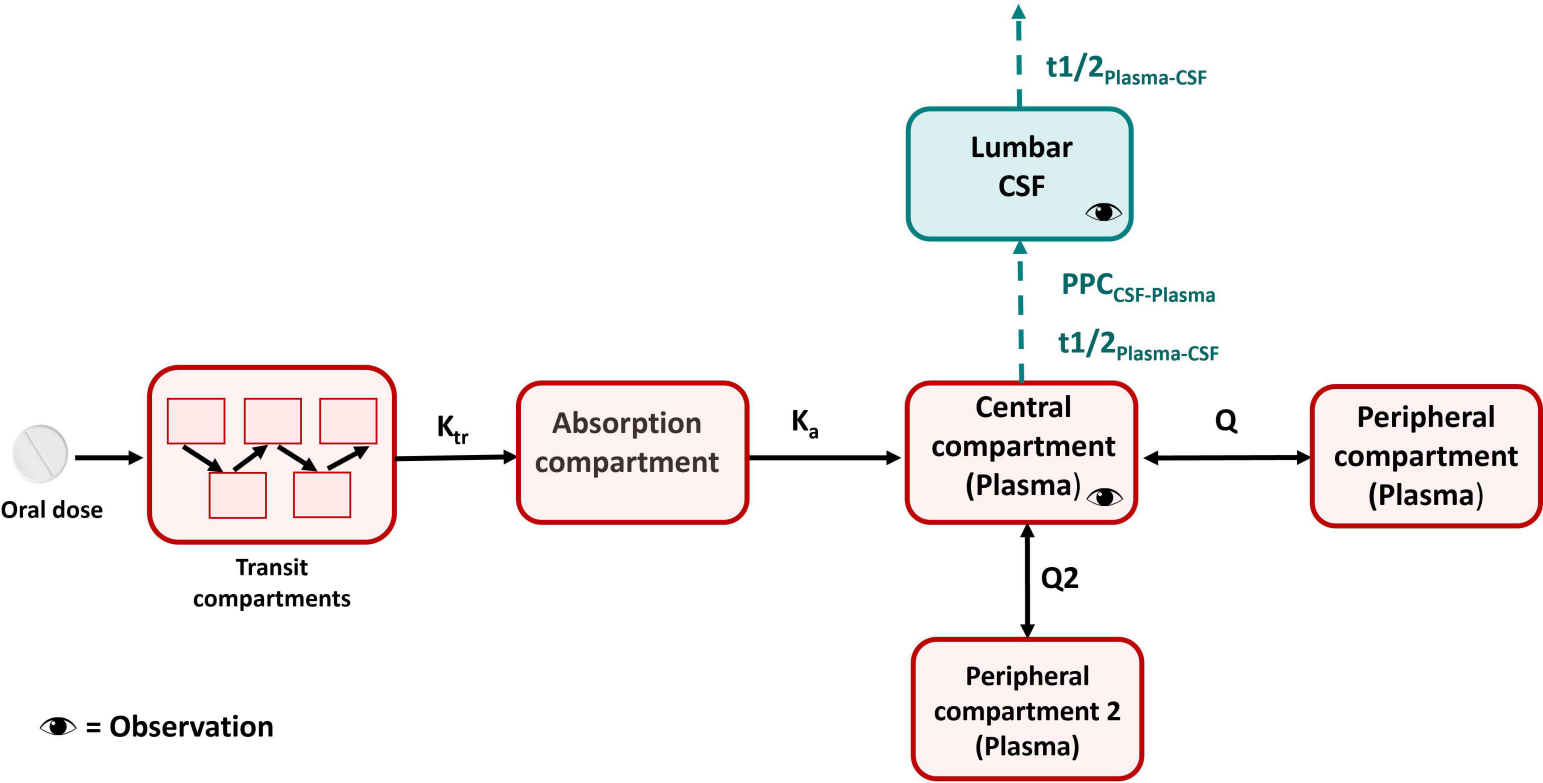
Schematic representation of the clofazimine final model. Schematic representation of the final clofazimine model. K_tr_ is the first-order rate constant for drug passage through transit compartments. Q and Q2 represent the intercompartmental clearances between the central and the two peripheral plasma compartments. t1/2_Plasma-CSF_ is the equilibration half-life between plasma and CSF, which describes how soon the change in plasma is reflected in the CSF. PPC_CSF-Plasma_ is the pseudo-partition coefficient which represents the relative drug exposure in CSF compared to plasma at steady state.

A schematic representation of the final model for clofazimine is provided in Fig. 4. The final pharmacokinetic parameters and the respective 95% confidence intervals are shown in Table S3. Model-derived AUC_0–24h_ and C_max_ values for clofazimine are depicted in Fig. 2B with a typical concentration–time profile in plasma and CSF in Fig. 3B. More results on population pharmacokinetic modeling of clofazimine can be found in the Supplemental material.

## DISCUSSION

We investigated the pharmacokinetics of terizidone and clofazimine in plasma and CSF in patients treated for drug-resistant pulmonary TB. The plasma PK was consistent with previous studies. Terizidone, measured as cycloserine, achieved high CSF exposure with rapid equilibration between compartments. By contrast, clofazimine demonstrated low CSF concentrations relative to plasma and a slow equilibration half-life. CSF:plasma ratios are often based on total concentrations (12). Since only the unbound drug is pharmacologically active and able to cross into the CNS, this approach can underestimate the potential of highly protein-bound drugs and misrepresent their therapeutic value. Here, both compounds were found to penetrate the CSF in expected concentrations, roughly similar to their protein-unbound concentrations in plasma. Furthermore, a limitation of prior publications is the reliance on pair-wise plasma and CSF samples to determine a penetration ratio. This does not account for the delay in CSF equilibrium compared to plasma. Here, this is overcome with the use of pharmacometric models and broad sampling windows for CSF to allow model-based prediction of CSF:plasma AUC. Beyond determining CSF penetration of two second-line TB drugs, this study demonstrates an acceptable and reproducible method for evaluating CSF drug exposure. The study provided sufficient precision to characterize CSF penetration for both drugs, suggesting that this approach is feasible with relatively small sample sizes with minimal confounding factors.

For cycloserine, 69% of the plasma concentration reached the CSF at steady state. This aligns with reports of an *in vitro* unbound fraction of 80% and a human study indicating 13% protein-bound cycloserine (range 4%–26%), though published data remain limited (21, 22). The current study is the first to investigate cycloserine CSF penetration following terizidone administration in either animal models or humans. Terizidone, a prodrug, rapidly hydrolyzes into terephthalaldehyde and two molecules of cycloserine on absorption, although the exact metabolic pathway is unknown (23). Cycloserine is known to have high penetration through both inflamed and intact meninges relative to plasma (15, 24). CSF concentrations and matched CSF:plasma ratios following 500 mg or 750 mg terizidone were consistent with prior reports of cycloserine 500 mg daily, whether administered as a single or divided dose (15, 24). Terizidone has fewer adverse effects with similar tolerability to cycloserine (25). Given comparable plasma and CSF exposure and once-daily dosing, terizidone may be the preferred drug.

The CSF concentration of clofazimine was approximately 0.13% of the plasma concentration, in line with the high protein binding, >85% in mice and 99% in humans (unpublished data reported via personal communication in references (26, 27). The penetration ratios remained nearly constant across the cohort, and the observed interindividual variability in CSF exposure is mostly driven by variability in plasma exposure, which reflects differences in time on treatment, body composition, and possibly adherence to treatment. This suggests that clofazimine (the unbound fraction) may penetrate well across the blood-CSF barrier. Its high protein binding requires sensitive techniques like LC-MS/MS and an appropriately low LLOQ for detection (15, 28). This technical barrier may have rendered clofazimine levels in the CSF undetectable in prior studies and made this the first study to quantify clofazimine CSF penetration in humans (20, 26, 27). While not done historically in the TB field, CSF-to-plasma exposure ratios should be corrected for protein binding in plasma; otherwise, the CSF penetration of highly protein-bound drugs, such as delamanid and bedaquiline, will be severely underestimated (11). Clofazimine is not currently recommended for use in TBM regimens. Its anti-inflammatory effects and activity on persisters may offer benefits despite the low concentration. In view of its long elimination half-life, the use of a loading dose would be needed for application in TBM (29).

Interpreting whether the observed CSF concentrations would be effective against *Mycobacterium tuberculosis* (*M.tb*) in individuals with TBM is very difficult. Pharmacokinetic targets are typically set based on efficacy outcomes and/or drug exposure in relation to minimum inhibitory concentrations (MIC). Such targets are not well established even for pulmonary TB, and the translation to TBM adds a further layer of complication. The MIC for cycloserine against *M. tb* in Löwenstein–Jensen medium was previously set to 30 mg/L but has since been withdrawn (23). A tentative epidemiologic cut-off total concentration value of 16 mg/L and a critical concentration of 32 mg/L have been suggested (30, 31). The majority of our participants did not achieve this critical concentration in CSF. For clofazimine, the MIC in plasma is 0.25 mg/L with a critical total concentration of 1 mg/L (32, 33). CSF concentrations were orders of magnitude below these thresholds. However, MICs are determined *in vitro* under conditions that do not reflect protein binding, cellular environment, or metabolic state of bacilli *in vivo*. In addition, it cannot be excluded that AUC/MIC is the relevant pharmacodynamic measure for cycloserine or clofazimine, rather than the time above MIC (T/MIC). Also, drug accumulation may differ substantially across tissues, and it is possible that CNS tissue concentrations exceed those measured in CSF. Interpretation of CSF concentrations in the context of MIC and high protein binding remains challenging. Are higher doses needed for TBM efficacy, and are critical concentrations established in protein-rich environments applicable to the CSF? Further investigation is needed to determine the clinical relevance of these MIC values.

Our study design demonstrated the ability to determine CSF penetration in a relatively healthy cohort, without the confounding effects of disease-related BBB and BCSFB disruption. The CSF:plasma albumin ratio is a surrogate marker of barrier integrity. Inflammation may compromise barrier function in the early stages of TBM, allowing higher CSF drug concentrations through passive diffusion. Our estimates of drug penetration are thus conservative. In the early stages of TBM, drug levels might be higher. This approach is easy to implement, requires minimal resources, carries low risk to participants, and can be nested within clinical drug trials while mitigating recruitment and ethical challenges of TBM studies (11). Our findings suggest that meaningful pharmacokinetic conclusions can be drawn from cohorts of this size. In this context, the use of previously established models enables the interpretation of plasma data to inform the modeling of CSF concentrations. This approach adjusts the estimation of CSF penetration for the delay in concentration equilibration between compartments, rather than relying solely on matched plasma–CSF samples. The study employed intensive pharmacokinetic sample collection and validated LC-MS/MS assays with very low limits of quantitation. The extended sampling window post-dose allowed for an accurate assessment of the pharmacokinetic profiles, as both cycloserine and clofazimine exhibit a prolonged T_max_. This addressed previous limitations where a 10-hour period left AUC assessment incomplete in 25% of participants (14).

This study also has several potential limitations. Only one CSF sample was collected per participant to limit the risk of post-LP headache, excluding assessment of longitudinal changes in CSF drug concentrations in a single participant over time. Also, samples were collected only from the second week of treatment, with no 24-hour post-dose CSF samples, limiting the ability to characterize delays in CSF concentration relative to plasma for long half-life drugs like clofazimine, as plasma profiles remained nearly flat during the observed period. Missing serum creatinine data prevented the full characterization of renal function and its impact on the drug clearance of cycloserine. We did not control for food intake, which may have introduced variability in plasma C_max_ and T_max_; however, this is unlikely to affect the interpretation of CSF:plasma ratios, which were the primary focus. Lastly, the study recruited participants with pulmonary TB, not TBM, which may affect the direct applicability of the results to TBM treatment. Meningeal inflammatory processes can alter BBB and BCSFB permeability, impacting drug pharmacokinetics during the first weeks of treatment. However, this study highlights a key gap in many TBM-focused pharmacokinetic studies. Little is known about CNS penetration later in treatment when the BBB and BCSFB have partially recovered. Our population’s intact barriers may be representative of later CNS penetration, as these are thought to recover over 2 weeks with effective treatment (10).

In conclusion, the high CSF penetration of terizidone/cycloserine supports its potential role in TBM treatment and underscores the need for its evaluation in future clinical trials. Clofazimine, despite its high protein binding, demonstrated CSF penetration, suggesting it may also warrant further investigation for CNS tuberculosis. This study establishes a feasible, low-risk, and reproducible method for screening CNS drug penetration, facilitating early-stage evaluation of pipeline drugs for application in TBM. We recommend incorporating similar evaluations into the development of novel anti-TB agents to optimize CNS drug selection and regimen design.

## SUPPLEMENTAL MATERIAL

Data S1 aac.00931-25-s0001.docx NONMEM code used for pharmacokinetic modeling.

Supplemental material aac.00931-25-s0002.docx Fig. S1 to S13; Tables S1 to S3.

ASM does not own the copyrights to Supplemental Material that may be linked to, or accessed through, an article. The authors have granted ASM a non-exclusive, world-wide license to publish the Supplemental Material files. Please contact the corresponding author directly for reuse.
