# Supplementary material for: Cerebrospinal fluid penetration of cycloserine/terizidone and clofazimine in patients with pulmonary TB
Source: Antimicrob Agents Chemother. 2025 Oct 21;69(12):e00931-25. doi: 10.1128/aac.00931-25 (PMC12691663; doi:10.1128/aac.00931-25)
Supplement: Data S1 — NONMEM code used for pharmacokinetic modeling. [file aac.00931-25-s0001.docx]

Supplement 2 to CSF Penetration of Terizidone and Clofazimine in Patients with Pulmonary TB, Upton 2025

**NONMEM code for cycloserine plasma model**

; Settings for the memory of NONMEM

$SIZES PD=-1000 LVR=-150 LTH=-200 MAXFCN=10000000 LNP4=-150000

;-----------------------------------------------------------------------------------------------------------------------------------------------

$PROBLEM -

;-----------------------------------------------------------------------------------------------------------------------------------------------

$INPUT -

;-----------------------------------------------------------------------------------------------------------------------------------------------

$DATA -

;------------------------------------------------------------------------------------------------------------------------------------------------

$ABB DERIV2=NO ; Prevents the computation of second derivatives, which are needed only for the Laplacian method.

;------------------------------------------------------------------------------------------------------------------------------------------------

$SUBROUTINE ADVAN13 TRANS1 TOL=9 ; TOL is the precision to solve differential equations

ATOL=9 ; absolute tolerance, 10^ATOL of your dose unit. A lower value makes the model run faster

;-------------------------------------------------------------------------------------------------------------------------------------------------

$MODEL

NCOMPARTMENTS = 2

COMP=(ABS DEFDOSE)

COMP=(CENTRAL DEFOBSERVATION)

;----------------------------------------------------------------------------------------------------------------------------------------------

$PK

;----Defining Between-Occasion Variability-----------------------------------------------------------------------------------------------------

BOVCL = 0

BOVBIO = 0

BOVKA = 0

BOVMTT = 0

IF(OCC==1) THEN

BOVCL = ETA(5)

BOVBIO = ETA(7)

BOVKA = ETA(9)

BOVMTT = ETA(11)

ENDIF

IF (OCC==2) THEN

BOVCL = ETA(6)

BOVBIO = ETA(8)

BOVKA = ETA(10)

BOVMTT = ETA(12)

ENDIF

;----Defining Between-Subject Variability-----------------------------------------------------------------------------------------------------

BSVCL = ETA(1)

BSVV = ETA(2)

BSVKA = ETA(3)

BSVBIO = ETA(4)

;----Covariates-----------------------------------------------------------------------------------------------------

; -------------- Calculation of Fat-free Mass-----------------------------------------------------------------------

;Conversion from cm to m--------------

HTM = HT/100

IF (SEX.EQ.1) THEN ; female

WHSMAX=37.99

WHS50=35.98

ELSE ;males

WHSMAX=42.92

WHS50=30.93

ENDIF

HTM2 = HTM**2

FFM = (WHSMAX*HTM2*WT)/(WHS50*HTM2+WT)

FAT = WT-FFM

IF (FAT.LT.0) FAT = 0

;------- Typical values of covariates

TVWT = 52

TVFAT = 13.019

TVFFM = 37.21

;--------- Allometric scaling and covariates

ALLMCL_WT = (WT/TVWT)**0.75

ALLMV_WT = (WT/TVWT)

ALLMCL_FAT = (FAT/TVFAT)**0.75

ALLMV_FAT = (FAT/TVFAT)

ALLMCL_FFM = (FFM/TVFFM)**0.75

ALLMV_FFM = (FFM/TVFFM)

;---------Typical values------------------------------------------------------------------------------------------------------------------------------------

TVCL = THETA(1)*ALLMCL_FFM*EXP(THETA(9)*(AGE-37))

TVV = THETA(2)*ALLMV_FFM

TVKA = THETA(3)

TVBIO = THETA(4)

TVMTT = THETA(7)

TVNN = THETA(8)

;-----------Define parameters-----------------------------------------------------------------------------------------------------------------------

CL = TVCL*EXP(BSVCL+BOVCL) ; CLEARANCE

V = TVV*EXP(BSVV) ; CENTRAL VOL.

KA = TVKA*EXP(BSVKA+BOVKA) ; ABS. RATE CONSTANT

BIO = TVBIO*EXP(BSVBIO+BOVBIO) ; BIOAVAILABILITY

MTT =TVMTT*EXP(BOVMTT) ; MTT TIME

NN = TVNN ; Number of transit compartments

;-----------------------------------------------------------------------------------------------------------------------------------------------------

; re-parameterization

K = CL/V ;(rate constant of elimination)

; Transit compartment absorption

F1=0 ; I need to set bioavailability in compartment 1 to 0 for this implementation of the transit compartment absorption

KTR = (NN+1)/MTT ; The number of actual transit compartments is NN+1, so this number can never be 0

IF (NEWIND/=2.OR.EVID>=3) THEN ; new individual, or reset event

; The values read here will be stored in TDOS and PD in this very PK call.

TNXD=TIME ; Time of the dose

PNXD=AMT ; Amount. If it's zero, the DE is deactivated.

ENDIF

TDOS=TNXD ; This will either save here the temporary values if it's a new individual...

PD=PNXD ; ...or the values which were read one record ahead during the execution of the previous record.

IF(AMT>0) THEN ; This reads one record ahead and stores the data to be used when running the following record

; IF(AMT.GT.0.AND.ALAG1.EQ.0) THEN ; Use this INSTEAD if there is ALAG, as it will also checks if the ALAG is not 0. Note that you normally do not want to include both ALAG and transit, this is a very exceptional case

TNXD=TIME

PNXD=AMT

ENDIF

; To speed up the computation, I calculate here all the non-time-varying quantities used in $DES

PIZZA = LOG(BIO*PD*KTR + 1E-12) - GAMLN(NN+1) ; without +0.00001, it won't work with ETAs in bioavailability

;--------Initializing the amount of drug in each compartment-----------------------------------------------------------------------------------------------

A_0(1) = 1E-12

A_0(2) = 1E-12

;----------------------------------------------------------------------------------------------------------------------------------------------------

$DES

TEMPO = T-TDOS ; this is time after dose for the transit, it should always be >= 0

KTT = 0

TRANSIT = 0

IF(PD.GT.0.AND.TEMPO.GT.0) THEN ; This happens only id PD>0, so only if a dose has been detected

KTT = KTR*(TEMPO)

TRANSIT = EXP(PIZZA+NN*LOG(KTT)-KTT)

ENDIF

DADT(1) = TRANSIT-KA*A(1)

DADT(2) = KA*A(1) -K*A(2)

;-------------------------------------------------------------------------------------------------------------------------------------------------------

$ERROR

IPRED=A(2)/V

LLOQ = 0.2 ; DEFINE YOUR OWN LLOQ HERE

CENS_THR = LLOQ

PROP = IPRED*THETA(5)

ADD = THETA(6)+(CENS_THR*0.2)

IF (ICALL/=4.AND.CENS==1) THEN

ADD = ADD +(CENS_THR*0.5)

ENDIF

NO_FIT = 0

IF (ICALL/=4.AND.CENS==2) THEN

PROP = 0

ADD = 10000000000

NO_FIT = 1

ENDIF

W = SQRT(ADD**2+PROP**2)

; Protective code

IF (W.LE.0.000001) W=0.000001

IRES=DV-IPRED

IWRES=IRES/W

Y = IPRED + W*ERR(1)

IF (ICALL==4.AND.Y<=CENS_THR) Y = CENS_THR/2

; To calculate time after dose.

IF(AMT>0) THEN

TIMEDOSE = TIME

AMOUNTDOSE = AMT

ENDIF

TAD = TIME-TIMEDOSE

VARCL = BSVCL + BOVCL

VARBIO = BSVBIO + BOVBIO

VARAUC = BSVBIO + BOVBIO - BSVCL - BOVCL

VARABS = BOVKA + BSVKA - BOVMTT

;------------------------------------------RETRIEVE AMOUNT IN EACH COMPARTMENT---------------------------------------------------------------------------------------

AA1 = A(1)

AA2 = A(2)

;--------------------------------------------------------------------------------------------------------------------------------------------------------------------

$THETA

(0, 0.544,90) ; 1 CL [L/h]

(0, 13.9,800) ; 2 V [L]

(0, 2.29,10) ; 3 KA [1/h]

(1) FIX ; 4 BIO

(0, 0.0762,1) ; 5 PROP []

(0, 0.136,5) ; 6 ADD [mg/L]

(0, 1.34,3) ; 7 MTT

(0, 2.38,20) ; 8 NN []

(-5, -0.0144,10) ; 9 AGE_CL

$OMEGA BLOCK(1) 0.0653 ; 1 BSV CL

$OMEGA BLOCK(1) 0 FIX ; 2 BSV V

$OMEGA BLOCK(1) 0 FIX ; 3 BSV KA

$OMEGA BLOCK(1) 0 FIX ; 4 BSV BIO

;---------------------------------------------------------------------------------------------------------------------------------------------------------------------

$OMEGA BLOCK(1) 0 FIX ; 5 BOVCL

$OMEGA BLOCK(1) SAME ; 6 BOVCL

;----------------------------------------------------------------------------------------------------------------------------------------------------------------------

$OMEGA BLOCK(1) 0.0299 ; 7 BOVBIO

$OMEGA BLOCK(1) SAME ; 8 BOVBIO

;---------------------------------------------------------------------------------------------------------------------------------------------------------------------

$OMEGA BLOCK(1) 0.908 ; 9 BOVKA

$OMEGA BLOCK(1) SAME ; 10 BOVKA

;---------------------------------------------------------------------------------------------------------------------------------------------------------------------

$OMEGA BLOCK(1) 0.288 ; 11 BOVMTT

$OMEGA BLOCK(1) SAME ; 12 BOVMTT

;--------------------------------------------------------------------------------------------------------------------------------------------------------

$SIGMA 1 FIX

;-------------------------------------------------------------------------------------------------------------------------------------------------------

$ESTIMATION MSFO=run547.msf MAXEVAL=9999 PRINT=1 METHOD=1 INTER NOABORT

NSIG=3 SIGL=9

NONINFETA=1 ETASTYPE=1

;-------------------------------------------------------------------------------------------------------------------------------------------------------

$TABLE –

The NONMEM code for the clofazimine plasma model is available in the supplementary materials of this publication: <https://doi.org/10.1093/jac/dkaa310>.

NONMEM code for cycloserine CSF modeling, with individual plasma parameters fixed from the plasma model.

; Settings for the memory of NONMEM

$SIZES PD=-1000 LVR=-150 LTH=-200 MAXFCN=10000000 LNP4=-150000

;-----------------------------------------------------------------------------------------------------------------------------------------------

$PROBLEM -

;-----------------------------------------------------------------------------------------------------------------------------------------------

$INPUT -

;-----------------------------------------------------------------------------------------------------------------------------------------------

$DATA -

;------------------------------------------------------------------------------------------------------------------------------------------------

$ABB DERIV2=NO ; Prevents the computation of second derivatives, which are needed only for the Laplacian method.

;------------------------------------------------------------------------------------------------------------------------------------------------

$SUBROUTINE ADVAN13 TRANS1 TOL=9 ; TOL is the precision to solve differential equations

ATOL=9

;-------------------------------------------------------------------------------------------------------------------------------------------------

$MODEL

NCOMPARTMENTS = 3

COMP=(ABS DEFDOSE)

COMP=(CENTRAL DEFOBSERVATION)

COMP=(CSF)

;----------------------------------------------------------------------------------------------------------------------------------------------

$PK

;----Defining Between-Occasion Variability-----------------------------------------------------------------------------------------------------

BSVPPC_CSF = ETA(1)

BSVKE0_CSF = ETA(2)

;-----Typical values for CSF------------------------------------------------------------------------------------------------------------------

TVPPC_CSF = THETA(3)

TVKE0_CSF = THETA(4)

;-----Define parameters plasma (These columns were included in the dataset after obtaining the individual pk parameter from the plasma model--

CL = ICL

V = IV

KA = IKA

BIO = IBIO

MTT = IMTT

NN = INN

;----Define parameters CSF---------------------------------------------------------------------------------------------------------------------

PPC_CSF = TVPPC_CSF*EXP(BSVPPC_CSF)

KE0_CSF = TVKE0_CSF*EXP(BSVKE0_CSF)

HL_CSF = LOG(2)/KE0_CSF

;-----------------------------------------------------------------------------------------------------------------------------------------------

; re-parameterization

K = CL/V ;(rate constant of elimination)

; Transit compartment absorption

F1=0 ; I need to set bioavailability in compartment 1 to 0 for this implementation of the transit compartment absorption

KTR = (NN+1)/MTT ; The number of actual transit compartments is NN+1, so this number can never be 0

IF (NEWIND/=2.OR.EVID>=3) THEN ; new individual, or reset event

; The values read here will be stored in TDOS and PD in this very PK call.

TNXD=TIME ; Time of the dose

PNXD=AMT ; Amount. If it's zero, the DE is deactivated.

ENDIF

TDOS=TNXD ; This will either save here the temporary values if it's a new individual...

PD=PNXD ; ...or the values which were read one record ahead during the execution of the previous record.

IF(AMT>0) THEN ; This reads one record ahead and stores the data to be used when running the following record

; IF(AMT.GT.0.AND.ALAG1.EQ.0) THEN ; Use this INSTEAD if there is ALAG, as it will also checks if the ALAG is not 0. Note that you normally do not want to include both ALAG and transit, this is a very exceptional case

TNXD=TIME

PNXD=AMT

ENDIF

; To speed up the computation, I calculate here all the non-time-varying quantities used in $DES

PIZZA = LOG(BIO*PD*KTR + 1E-12) - GAMLN(NN+1) ; without +0.00001, it won't work with ETAs in bioavailability

;;;--------------------------------------------------------------

A_0(1) = 1E-12

A_0(2) = 1E-12

A_0(3) = 1E-12

;--------Initializing the amount of drug in each compartment--------------------------------------------------------------------------------------

$DES

C2 = A(2)/V

TEMPO = T-TDOS ; this is time after dose for the transit, it should always be >= 0

KTT = 0

TRANSIT = 0

IF(PD.GT.0.AND.TEMPO.GT.0) THEN ; This happens only id PD>0, so only if a dose has been detected

KTT = KTR*(TEMPO)

TRANSIT = EXP(PIZZA+NN*LOG(KTT)-KTT)

ENDIF

DADT(1) = TRANSIT-KA*A(1)

DADT(2) = KA*A(1) -K*A(2)

DADT(3) = KE0_CSF*(PPC_CSF*C2 - A(3))

;-------------------------------------------------------------------------------------------------------------------------------------------------------

$ERROR

LLOQ_P = 0.16 ; LLOQ plasma assay

LLOQ_E = 0.2 ; LLOQ CSF assay

CENS_THR_P = LLOQ_P

CP = A(2)/V

CE = A(3)

IPRED_P = CP

PROP_P = IPRED_P*THETA(1)

ADD_P = THETA(2)+(CENS_THR_P*0.2)

IF (ICALL/=4.AND.CENS==1.AND.DVID==1) THEN

ADD_P = ADD_P +(LLOQ_P*0.5)

ENDIF

IF (ICALL/=4.AND.CENS==2.AND.DVID==1) THEN

PROP_P = 0

ADD_P = 10000000000

NO_FIT = 1

ENDIF

W_P= SQRT((ADD_P)**2+(PROP_P)**2)

CENS_THR_E = LLOQ_E

IPRED_E = CE

PROP_E = IPRED_E*THETA(5)

ADD_E = THETA(6) + (0.2*CENS_THR_E)

IF(ICALL/=4.AND.CENS==1.AND.DVID==2) THEN

ADD_E = ADD_E + (LLOQ_E*0.5)

ENDIF

IF (ICALL/=4.AND.CENS==2.AND.DVID==2) THEN

PROP_E = 0

ADD_E = 10000000000

NO_FIT = 1

ENDIF

W_E = SQRT((ADD_E)**2 + (PROP_E)**2)

ERROR_P = W_P * ERR(1)

ERROR_E = W_E * ERR(1)

;Redefine IPRED & weighting

IPRED = IPRED_P

W = W_P

ERROR_TERM = ERROR_P

IF(DVID==2) THEN

IPRED = IPRED_E

W = W_E

ERROR_TERM = ERROR_E

ENDIF

; Protective code

IF (W.LE.0.000001) W=0.000001

IRES=DV-IPRED

IWRES=IRES/W

Y = IPRED + ERROR_TERM

; To prevent simulation (ICALL==4) of negative values. It set a positive lower bound for Y, so that VPCs in the log-scale can be plotted

IF (DVID==1.AND.ICALL==4.AND.Y<=LLOQ_P) Y=LLOQ_P/2

IF (DVID==2.AND.ICALL==4.AND.Y<=LLOQ_E) Y=LLOQ_E/2

; To calculate time after dose.

IF(AMT>0) THEN

TIMEDOSE = TIME

AMOUNTDOSE = AMT

ENDIF

TAD = TIME-TIMEDOSE

;------------------------------------------RETRIEVE AMOUNT IN EACH COMPARTMENT---------------------------------------------------------------------------------------

AA1 = A(1)

AA2 = A(2)

AA3 = A(3)

;--------------------------------------------------------------------------------------------------------------------------------------------------------------------

$THETA

(0) FIX ; 1 PROP []

(0) FIX ; 2 ADD [mg/L]

(0, 0.692,1.5) ; 3 PPC_CSF [.]

(0, 0.149,10) ; 4 KE0_CSF [1/h]

(0, 0.255,1) ; 5 PROP_CSF [%]

(0) FIX ; 6 ADD_CSF [mg/L]

$OMEGA BLOCK(1) 0 FIX ; 1 BSVPPC_CSF

$OMEGA BLOCK(1) 0 FIX ; 2 BSVKE0_CSF

;-------------------------------------------------------------------------------------------------------------------------------------------------------------------

$SIGMA 1 FIX

;-------------------------------------------------------------------------------------------------------------------------------------------------------

$ESTIMATION MSFO=run54.msf MAXEVAL=9999 PRINT=1 METHOD=1 INTER NOABORT

NSIG=3 SIGL=9

NONINFETA=1 ETASTYPE=1

;-------------------------------------------------------------------------------------------------------------------------------------------------------

$TABLE –

NONMEM code for clofazimine CSF modeling, with individual plasma parameters fixed from the plasma model.

; Settings for the memory of NONMEM

$SIZES PD=-1000 LVR=-150 LTH=-200 MAXFCN=10000000 LNP4=-150000

;-----------------------------------------------------------------------------------------------------------------------------------------------

$PROBLEM -

;-----------------------------------------------------------------------------------------------------------------------------------------------

$INPUT -

;-----------------------------------------------------------------------------------------------------------------------------------------------

$DATA -

;------------------------------------------------------------------------------------------------------------------------------------------------

$ABB DERIV2=NO ; Prevents the computation of second derivatives, which are needed only for the Laplacian method.

;------------------------------------------------------------------------------------------------------------------------------------------------

$SUBROUTINE ADVAN13 TRANS1 TOL=9 ; TOL is the precision to solve differential equations

ATOL=9 ; absolute tolerance, 10^ATOL of your dose unit. A lower value makes the model run faster

;-------------------------------------------------------------------------------------------------------------------------------------------------

$MODEL

NCOMPARTMENTS = 5

COMP=(ABS DEFDOSE)

COMP=(CENTRAL DEFOBSERVATION)

COMP=(PERI1)

COMP=(PERI2)

COMP=(CSF)

;----------------------------------------------------------------------------------------------------------------------------------------------

$PK

;----Defining Between-Occasion Variability-----------------------------------------------------------------------------------------------------

BSVPPC_CSF = ETA(1)

BSVKE0_CSF = ETA(2)

;-----Typical values for CSF------------------------------------------------------------------------------------------------------------------

TVPPC_CSF = THETA(3)

TVKE0_CSF = THETA(4)

;-----Define parameters plasma----------------------------------------------------------------------------------------------------------------

CL = ICL ; CLEARANCE

V = IV7 ; CENTRAL VOL.

KA = IKA ; ABS. RATE CONSTANT

BIO = IBIO ; BIOAVAILABILITY

MTT = IMTT ; MTT TIME

V3 = IV8 ; PERIPH VOL

Q = IQ ; INTER COMPT CL

V4 = IV9 ; PERIPH VOL2

Q2 = IQ2 ; INTER COMPT CL2

NN = INN ; Number of transit compartments

;----Define parameters CSF---------------------------------------------------------------------------------------------------------------------

PPC_CSF = TVPPC_CSF*EXP(BSVPPC_CSF)

KE0_CSF = TVKE0_CSF*EXP(BSVKE0_CSF)

HL_CSF = LOG(2)/KE0_CSF

;----------------------------------------------------------------------------------------------------------------------------------------------

; re-parameterization

K = CL/V ;(rate constant of elimination)

K23 = Q/V ; (rate constant from central to peripheral 1)

K32 = Q/V3 ;(rate constant from peripheral 1 to central)

K24 = Q2/V ;(rate constant from central to peripheral 2)

K42 = Q2/V4 ; (rate constant from peripheral 2 to central)

;----------------------------------------------------------------------------------------------------------------------------------------------

; Transit compartment absorption

F1=0 ; I need to set bioavailability in compartment 1 to 0 for this implementation of the transit compartment absorption

KTR = (NN+1)/MTT ; The number of actual transit compartments is NN+1, so this number can never be 0

IF (NEWIND/=2.OR.EVID>=3) THEN ; new individual, or reset event

; The values read here will be stored in TDOS and PD in this very PK call.

TNXD=TIME ; Time of the dose

PNXD=AMT ; Amount. If it's zero, the DE is deactivated.

ENDIF

TDOS=TNXD ; This will either save here the temporary values if it's a new individual...

PD=PNXD ; ...or the values which were read one record ahead during the execution of the previous record.

IF(AMT>0) THEN ; This reads one record ahead and stores the data to be used when running the following record

; IF(AMT.GT.0.AND.ALAG1.EQ.0) THEN ; Use this INSTEAD if there is ALAG, as it will also checks if the ALAG is not 0. Note that you normally do not want to include both ALAG and transit, this is a very exceptional case

TNXD=TIME

PNXD=AMT

ENDIF

; To speed up the computation, I calculate here all the non-time-varying quantities used in $DES

PIZZA = LOG(BIO*PD*KTR + 1E-12) - GAMLN(NN+1) ; without +0.00001, it won't work with ETAs in bioavailability

;--------Initializing the amount of drug in each compartment----------------------------------------------------------------------------------------

A_0(1) = 1E-12

A_0(2) = 1E-12

A_0(3) = 1E-12

A_0(4) = 1E-12

A_0(5) = 1E-12

;----------------------------------------------------------------------------------------------------------------------------------------------------

$DES

C2 = A(2)/V

TEMPO = T-TDOS ; this is time after dose for the transit, it should always be >= 0

KTT = 0

TRANSIT = 0

IF(PD.GT.0.AND.TEMPO.GT.0) THEN ; This happens only id PD>0, so only if a dose has been detected

KTT = KTR*(TEMPO)

TRANSIT = EXP(PIZZA+NN*LOG(KTT)-KTT)

ENDIF

DADT(1) = TRANSIT-KA*A(1)

DADT(2) = KA*A(1)-K*A(2)-K23*A(2)+K32*A(3) -K24*A(2)+K42*A(4)

DADT(3) = K23*A(2)-K32*A(3)

DADT(4) = K24*A(2)-K42*A(4)

DADT(5) = KE0_CSF*(PPC_CSF*C2 - A(5))

;-------------------------------------------------------------------------------------------------------------------------------------------------------

$ERROR

LLOQ_P = 0.00781 ; LLOQ plasma assay

LLOQ_E = 0.00005 ; LLOQ CSF assay

CENS_THR_P = LLOQ_P

CP = A(2)/V

CE = A(5)

IPRED_P = CP

PROP_P = IPRED_P*THETA(1)

ADD_P = THETA(2)+(CENS_THR_P*0.2)

IF (ICALL/=4.AND.CENS==1.AND.DVID==1) THEN

ADD_P = ADD_P +(LLOQ_P*0.5)

ENDIF

IF (ICALL/=4.AND.CENS==2.AND.DVID==1) THEN

PROP_P = 0

ADD_P = 10000000000

NO_FIT = 1

ENDIF

W_P= SQRT((ADD_P)**2+(PROP_P)**2)

CENS_THR_E = LLOQ_E

IPRED_E = CE

PROP_E = IPRED_E*THETA(5)

ADD_E = THETA(6) + (0.2*CENS_THR_E)

IF(ICALL/=4.AND.CENS==1.AND.DVID==2) THEN

ADD_E = ADD_E + (LLOQ_E*0.5)

ENDIF

IF (ICALL/=4.AND.CENS==2.AND.DVID==2) THEN

PROP_E = 0

ADD_E = 10000000000

NO_FIT = 1

ENDIF

W_E = SQRT((ADD_E)**2 + (PROP_E)**2)

ERROR_P = W_P * ERR(1)

ERROR_E = W_E * ERR(1)

;Redefine IPRED & weighting

IPRED = IPRED_P

W = W_P

ERROR_TERM = ERROR_P

IF(DVID==2) THEN

IPRED = IPRED_E

W = W_E

ERROR_TERM = ERROR_E

ENDIF

; Protective code

IF (W.LE.0.000001) W=0.000001

IRES=DV-IPRED

IWRES=IRES/W

Y = IPRED + ERROR_TERM

IF (DVID==1.AND.ICALL==4.AND.Y<=LLOQ_P) Y=LLOQ_P/2

IF (DVID==2.AND.ICALL==4.AND.Y<=LLOQ_E) Y=LLOQ_E/2

; To calculate time after dose.

IF(AMT>0) THEN

TIMEDOSE = TIME

AMOUNTDOSE = AMT

ENDIF

TAD = TIME-TIMEDOSE

;------------------------------------------RETRIEVE AMOUNT IN EACH COMPARTMENT---------------------------------------------------------------------------------------

AA1 = A(1)

AA2 = A(2)

AA3 = A(3)

AA4 = A(4)

AA5 = A(5)

;--------------------------------------------------------------------------------------------------------------------------------------------------------------------

$THETA

(0) FIX ; 1 PROP []

(0) FIX ; 2 ADD [mg/L]

(0, 0.0013,1.5) ; 3 PPC_CSF [.]

(0, 0.0125,80) ; 4 KE0_CSF [1/h]

(0, 0.342,1) ; 5 PROP_CSF [%]

(0) FIX ; 6 ADD_CSF [mg/L]

$OMEGA BLOCK(1) 0 FIX ; 1 BSVPPC_CSF

$OMEGA BLOCK(1) 0 FIX ; 2 BSVKE0_CSF

;-------------------------------------------------------------------------------------------------------------------------------------------------------------------

$SIGMA 1 FIX

;-------------------------------------------------------------------------------------------------------------------------------------------------------

$ESTIMATION MSFO=run377.msf MAXEVAL=9999 PRINT=1 METHOD=1 INTER NOABORT

NSIG=3 SIGL=9

NONINFETA=1 ETASTYPE=1

;-------------------------------------------------------------------------------------------------------------------------------------------------------

$TABLE -
