# Supplementary material for: Cerebrospinal fluid penetration of cycloserine/terizidone and clofazimine in patients with pulmonary TB
Source: Antimicrob Agents Chemother. 2025 Oct 21;69(12):e00931-25. doi: 10.1128/aac.00931-25 (PMC12691663; doi:10.1128/aac.00931-25)
Supplement: Supplemental material — Fig. S1 to S13; Tables S1 to S3. [file aac.00931-25-s0002.docx]

Supplement to CSF Penetration of Terizidone and Clofazimine in Patients with Pulmonary TB, Upton 2025

**Contents**

[S1: Pharmacokinetic Profiles and Non-compartmental Analysis 2](#_Toc205537224)

[S2 Population PK Model 6](#_Toc205537226)

[S3: LC-MS/MS Assay methods and validation parameters 16](#_Toc205537227)

Note: Pharmacokinetic data are presented in both ng/mL and mg/L within this supplement. This reflects the original analytical workflows: non-compartmental analyses were conducted in ng/mL, while model-based pharmacometric analyses were performed in mg/L. To maintain consistency within each approach and preserve the integrity of the output files, units have been retained as originally generated.

# S1: Pharmacokinetic Profiles and Non-compartmental Analysis

**Table S1: Results of non-compartmental analysis of analytes presented as geometric mean (SD) for plasma.**

| Analyte | Cmax  ng/mL | Tmax  hours | AUCtau h*ng/mL | T1/2  hours |
| --- | --- | --- | --- | --- |
| Clofazimine | 486 (1.7) | 5.0 (1.7) | 9694 (1.6) | 68.7 (2.1) |
| Cycloserine 500 mg | 49 154 (1.2) | 3.6 (1.7) | 804 478 (1.3) | 17.8 (1.4) |
| Cycloserine 750 mg | 51 476 (1.3) | 4.3 (1.6) | 838 089 (1.5) | 18.7 (1.4) |

**Table S2: Model-predicted plasma pharmacokinetic parameters for a typical individual in the cohort.**

| Analyte | Cmax  ng/mL | Tmax  hours | AUCtau h*ng/mL | T1/2  hours |
| --- | --- | --- | --- | --- |
| Clofazimine | 502 (1.6) | 5.5 (1.4) | 9860 (1.6) | 107 |
| Cycloserine 500 mg | 50 169 (1.2) | 3.6 (1.7) | 809 613 (1.3) | 16.2 (1.3) |
| Cycloserine 750 mg | 52 687 (1.3) | 4.2 (1.6) | 844 225 (1.4) | 17.5 (1.4) |

Note: non-compartmental analysis has limitations in estimating T1/2 of a drug with a long elimination half-life when data are sampled up to only 24 h post dose. The model-predicted T1/2 of clofazimine is a more robust and reliable estimate.


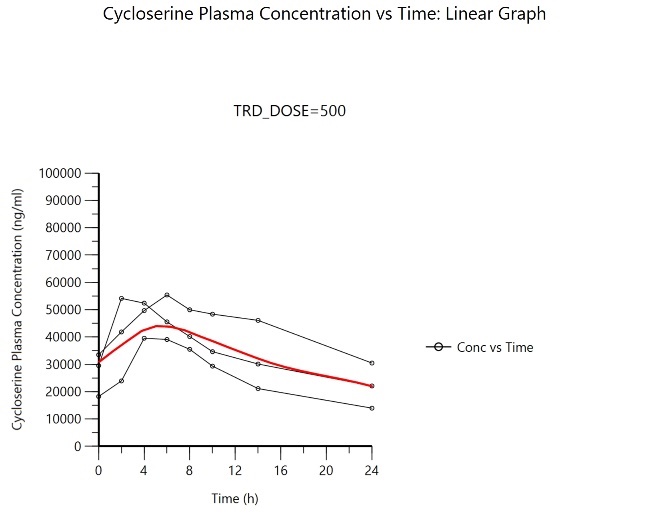

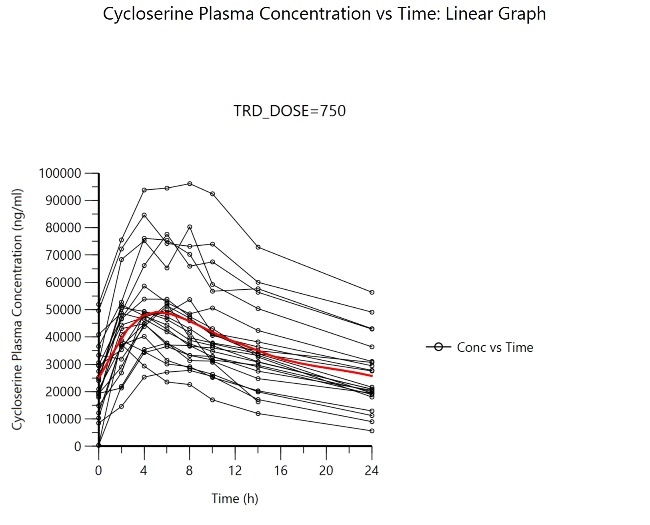


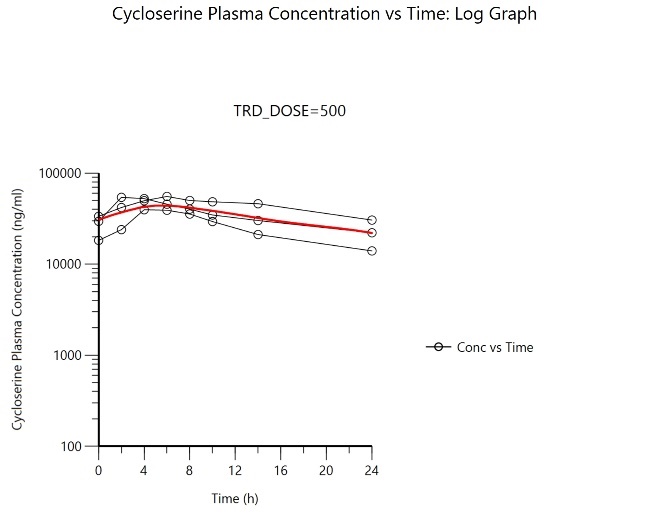

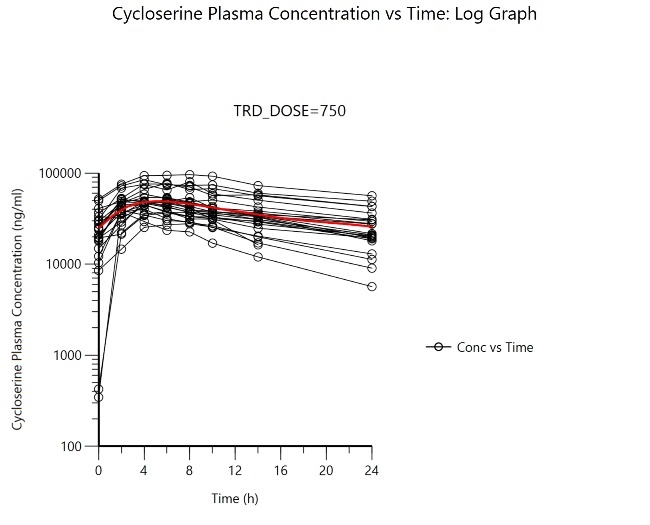


**Figure S1: Cycloserine concentration versus time profiles in plasma grouped by participant ID on the linear (top) and log (bottom) scale**. Terizidone 500 mg group on the left, and 750 mg group on the right. Individual observed plasma concentrations over time (black) with a locally smoothed curve (LOESS) illustrating overall trends. Each dot represents an individual measurement.


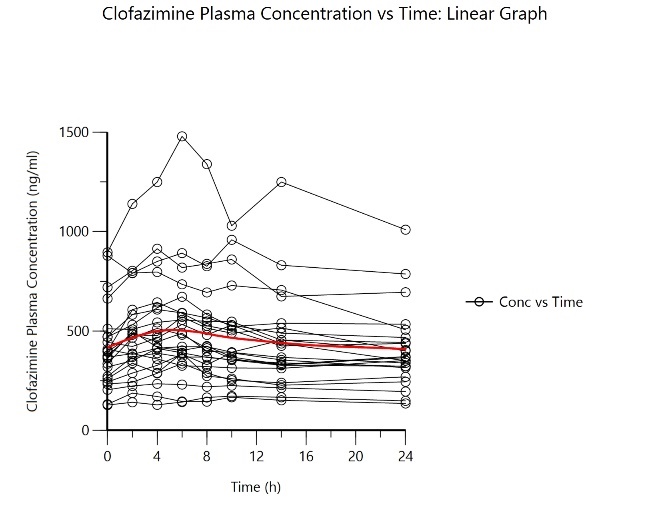

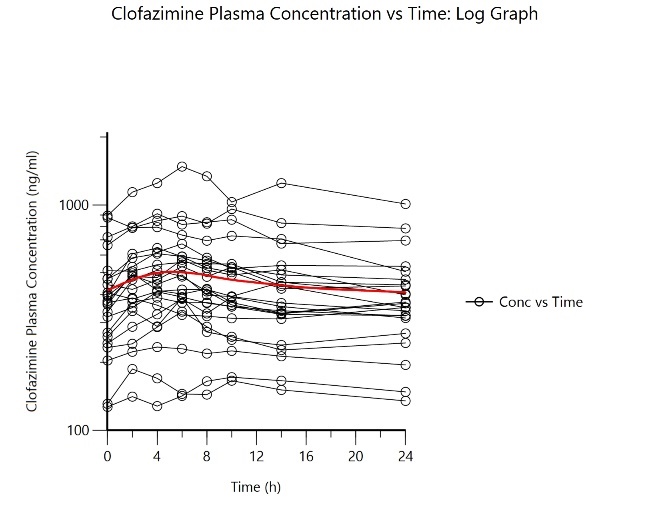


**Figure S2: Clofazimine concentration versus time profiles in plasma grouped by participant ID on the linear (left) and log (right) scale**. Individual observed plasma concentrations over time (black) with a locally smoothed curve (LOESS) illustrating overall trends. Each dot represents an individual measurement.

Plasma and CSF concentrations collected from the same participant at the same time (time-matched pairs) were plotted against each other, **Figure S3 and S4**. The figures demonstrate the relationship between drug found in CSF and plasma concentrations.

These have been included to show the raw data, the results shown in the box plots are direct time-matched pairs of CSF and plasma samples. The displayed ratio does not take into account the delay in equilibrium that is captured in the model results.


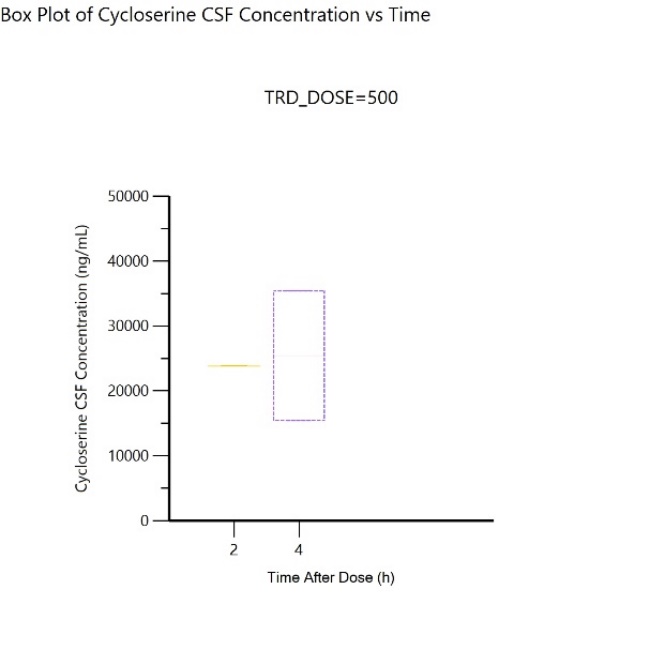

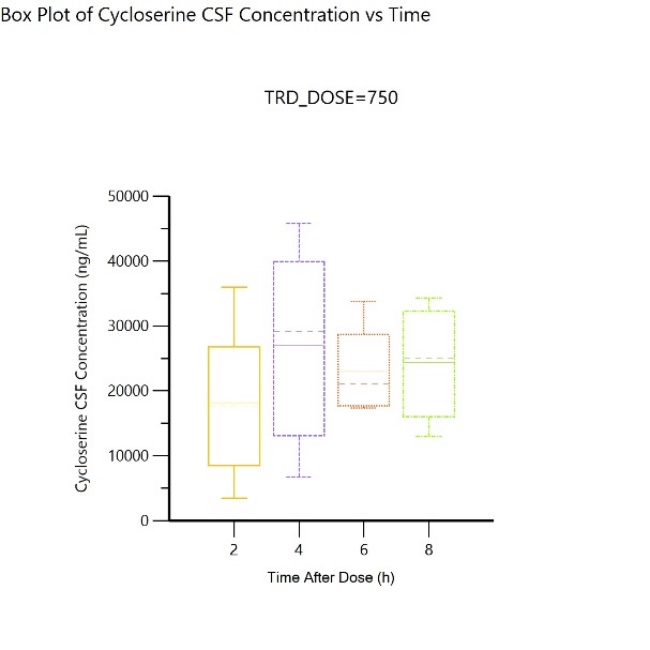

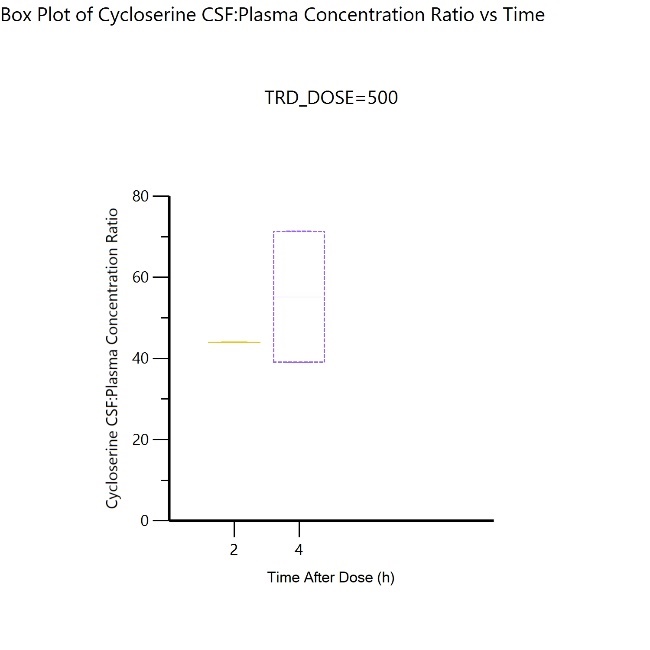

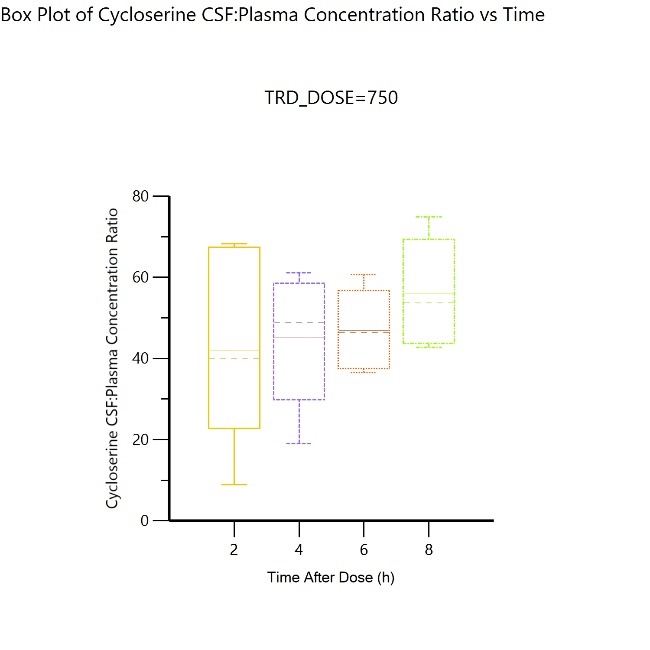


**Figure S3: Box Plot of cycloserine concentration versus time profiles in CSF (top) and CSF:Plasma ratio vs time (bottom).** Terizidone 500 mg group on the left, and 750 mg group on the right.


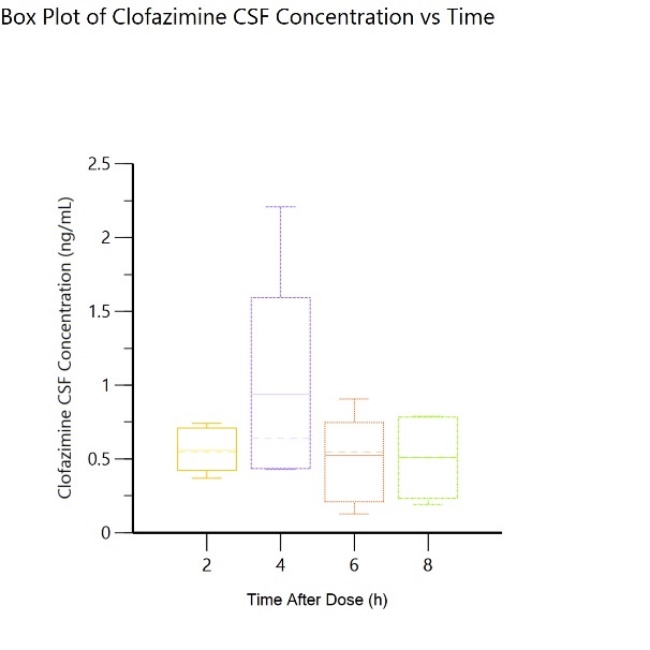

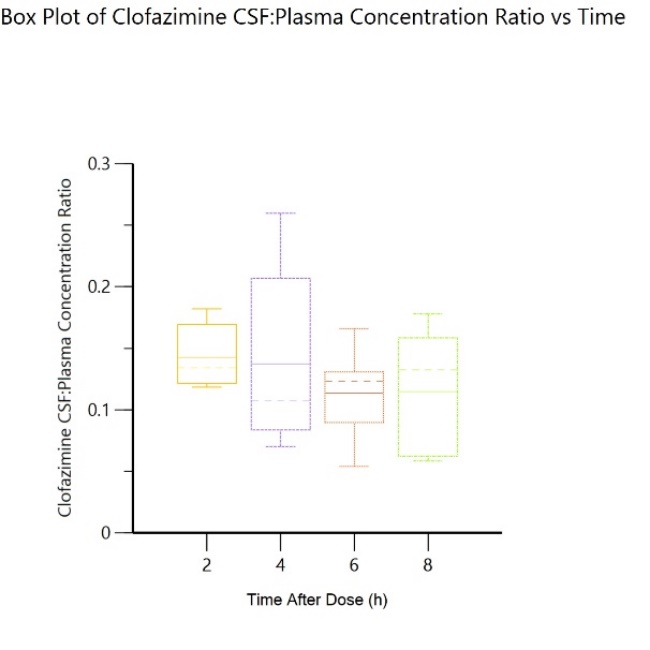


**Figure S4: Box Plot of clofazimine concentration versus time profiles in CSF (left) and CSF:Plasma concentration vs time ratio (right).** Clofazimine shows a high variability likely due to its unique distribution and long half-life in patients, with variable treatment duration.

**Figure S5: Scatter plot of matched CSF and plasma concentrations per participant for cycloserine (left) and clofazimine (right).**


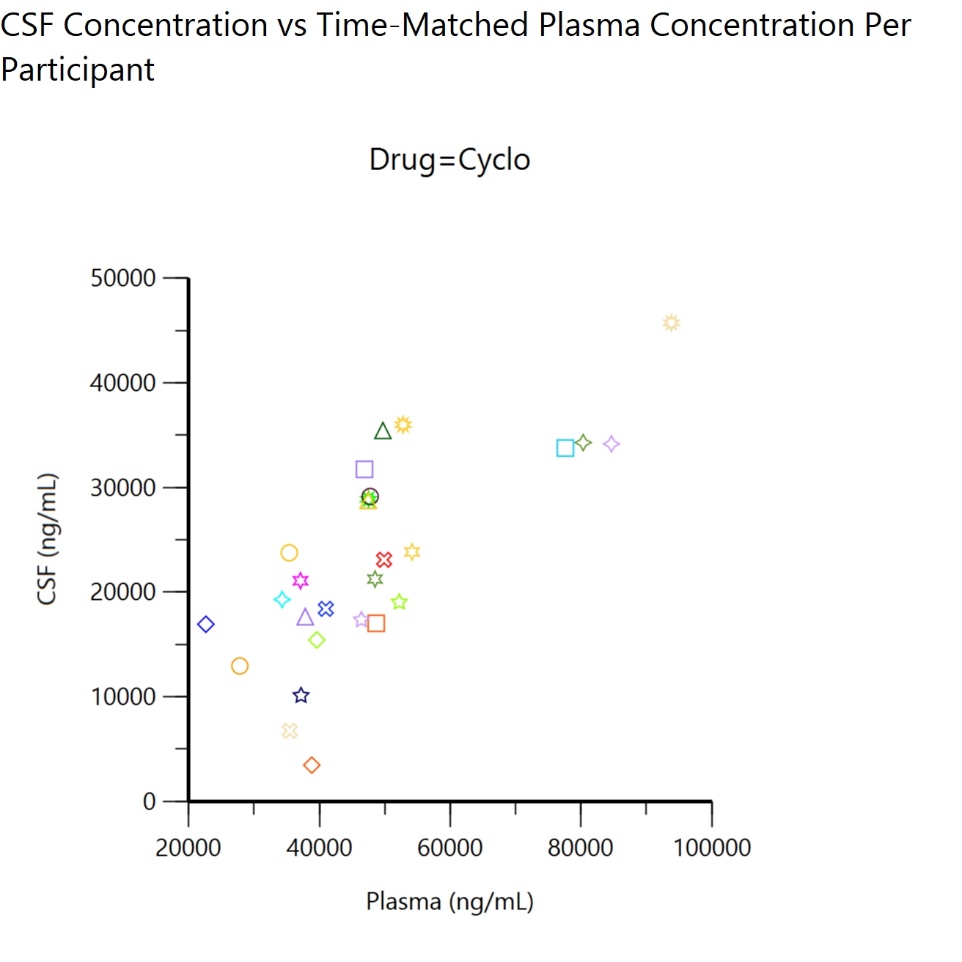

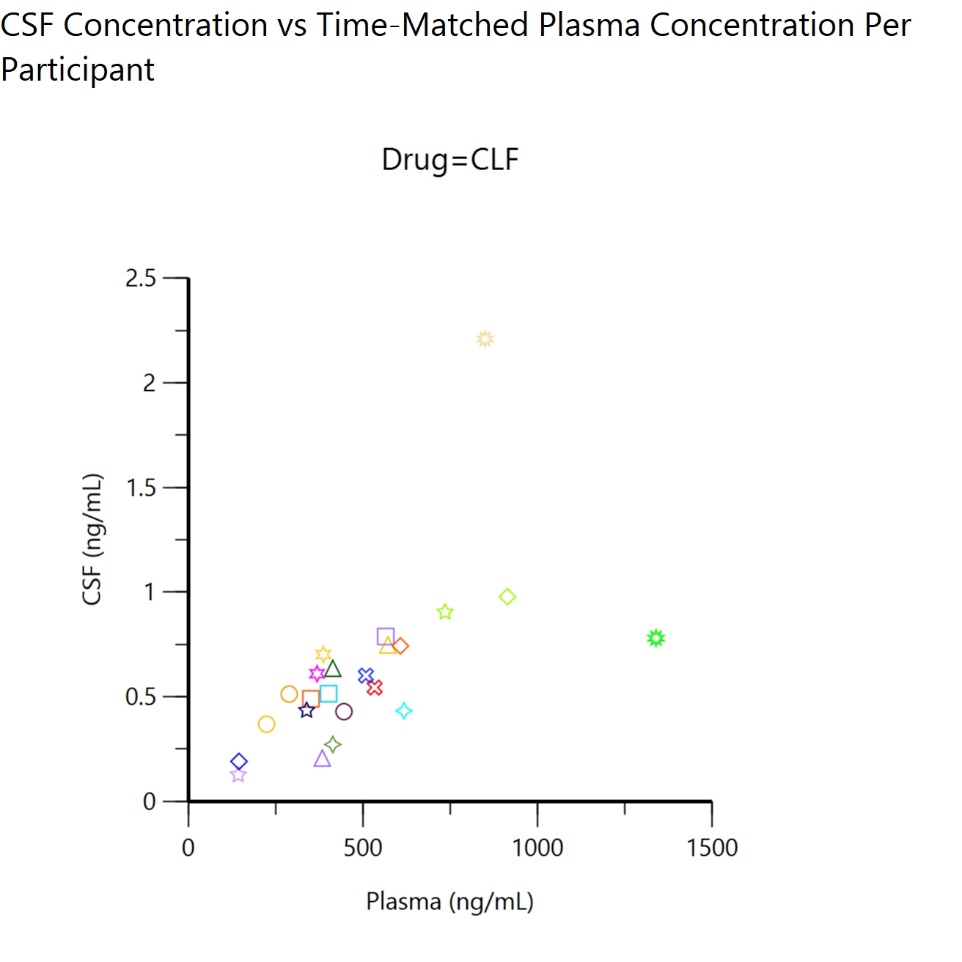


# S2 Population PK Model

The pharmacokinetic data was interpreted using nonlinear mixed-effects modelling in NONMEM 7.5 (REF) and the first-order conditional estimation with interaction (FOCE-I) algorithm. Population pharmacokinetic models were developed separately for cycloserine and clofazimine. A sequential (non-simultaneous) approach was used, first characterizing plasma drug concentrations and then incorporating CSF data(1).

For cycloserine, one- and two-compartment disposition models were tested with first-order absorption (with or without lag time or chain of transit compartments) and first-order elimination. The dose of terizidone was converted to the equivalent dose of cycloserine by assuming a 2:1 ratio of cycloserine to terizidone molecules and adjusting for the molecular weights (302.28 g/mol for terizidone and 102.09 g/mol for cycloserine)(2). This means that a 750 mg dose of terizidone was equivalent to a 506 mg dose of cycloserine. For clofazimine, a previously reported 3-compartment disposition model (3) was adapted and fit to the current data, since the known complex plasma pharmacokinetics and the limited plasma data collected in the current study did not support the creation of a model *de novo.*

Random effects on pharmacokinetic parameters were included if statistically significant, using a log-normal distribution(4). Between-subject variability (BSV) was explored for disposition parameters, and between-occasion variability (BOV) was explored for bioavailability and absorption parameters, with an occasion defined as a dosing event and its subsequent observations. Residual unexplained variability (RUV) was modelled using both additive and proportional error components, with the additive component constrained to be at least 20% of the assay’s LLOQ. The precision of the final model’s parameter estimates was assessed using the sampling importance resampling (SIR) method(5).

Allometric scaling based on total body weight or fat-free mass (FFM, estimated using the formula by Janmahasatian et al.(6)) was used to account for the influence of body size on drug disposition parameters. The exponents for allometric scaling of clearance and volume were fixed to 0.75 and 1, respectively(7). The effect of covariates such as age, HIV serostatus, concomitant antiretroviral treatment, smoking status, and time on treatment, was tested on the plasma pharmacokinetic parameters. The effect of CSF albumin and the CSF/plasma albumin ratio on CSF pharmacokinetic parameters was also assessed.

The individual plasma pharmacokinetic parameters were fixed, and the CSF concentrations were modelled implementing a hypothetical effect compartment linked to the central (plasma) compartment(8). This approach assumes negligible mass transfer from the central to the effect compartment and estimates the plasma-to-CSF equilibrium half-life (t1/2_Plasma-CSF_) and the CSF-to-plasma pseudo-partition coefficient (PPC_CSF-Plasma_). These parameters characterize the delay in concentration equilibration between the two compartments and the relative drug exposure in CSF compared to plasma at steady state, respectively.

The presence of an effect of CSF albumin or the CSF/plasma albumin ratio was assessed on CSF pharmacokinetic parameters.

The likelihood ratio test based on the drop in objective function value (OFV) was used to compare nested models, assuming an approximate χ2 distribution with degrees of freedom (n) equal to the number of additional estimated parameters. A stepwise approach was employed for model development, with a p-value of 0.05 for parameter inclusion and 0.01 for retention. Model development decisions were guided by statistical significance, physiological plausibility, clinical relevance, and diagnostic plots, including visual predictive checks (VPCs).

The final models were used to calculate the individual values area under the concentration-time curve from 0 to 24 hours post-dose (AUC_0-24h_) and the maximum concentration (C_max_) for both drugs in plasma and CSF. Descriptive statistics were used to summarize the demographic and pharmacokinetic parameters.

The value of free fraction of each drug was obtained from previous published protein binding data, to adjust the PPC by calculating the CSF to unbound plasma ratio

References

1. Zhang L, Beal SL, Sheiner LB. Simultaneous vs. Sequential Analysis for Population PK/PD Data I: Best-case Performance. J Pharmacokinet Pharmacodyn. 2003 Dec;30(6):387–404.

2. Zítková L, Toušek J. Pharmacokinetics of Cycloserine and Terizidone: A Comparative Study. Vol. 20, Chemotherapy. 1974. p. 18–28.

3. Abdelwahab MT, Wasserman S, Brust JCM, Gandhi NR, Meintjes G, Everitt D, et al. Clofazimine pharmacokinetics in patients with TB: dosing implications. Journal of Antimicrobial Chemotherapy. 2020;1–9.

4. Mould DR, Upton RN. Basic concepts in population modeling, simulation, and model-based drug development - Part 2: Introduction to pharmacokinetic modeling methods. CPT Pharmacometrics Syst Pharmacol. 2013;2(4).

5. Dosne AG, Bergstrand M, Karlsson MO. An automated sampling importance resampling procedure for estimating parameter uncertainty. J Pharmacokinet Pharmacodyn. 2017;44(6):509–20.

6. Janmahasatian S, Duffull SB, Ash S, Ward LC, Byrne NM, Green B. Quantification of lean bodyweight. Clin Pharmacokinet. 2005;44(10):1051–65.

7. Anderson BJ, Holford NHG. Mechanism-based concepts of size and maturity in pharmacokinetics. Annu Rev Pharmacol Toxicol. 2008;48:303–32.

8. Sheiner LB, Stanski DR, Vozeh S, Miller RD, Ham J. Simultaneous modeling of pharmacokinetics and pharmacodynamics: Application to d-tubocurarine. Clin Pharmacol Ther. 1979;25(3):358–71.

**Table S3. Final pharmacokinetic parameters estimate for cycloserine and clofazimine.**

|  | **Typical values (95% CI) ^a^** | |
| --- | --- | --- |
| **Parameter (units)** | **Cycloserine** | **Clofazimine** ^b^ |
| Clearance, CL (L/h) | 0.54 (0.488–0.609) ^c^ | 11.1 Fixed ^b, d^ |
| Central volume of distribution, Vc (L) | 13.9 (13.0–14.9) ^c^ | 240 Fixed ^b, e^ |
| Intercompartmental clearance, Q1 (L/h) | - | 54.7 Fixed ^b, d^ |
| Peripheral volume of distribution 1, Vp1 (L) | - | 12100 Fixed ^b^ |
| Intercompartmental clearance, Q2 (L/h) | - | 83.5 Fixed ^b, d^ |
| Peripheral volume of distribution 2, Vp2 (L) | - | 864 Fixed ^b, d^ |
| Bioavailability, F (.) | 1 Fixed | 1 Fixed ^b^ |
| Mean absorption transit time, MTT (h) | 1.34 (0.950–1.85) | 1.41 Fixed ^b^ |
| Number of absorption transit compartments, NN (.) | 2.38 (1.13–4.46) | 4.75 Fixed ^b^ |
| First-order absorption rate constant. Ka (h^-1^) | 2.29 (1.15–4.82) | 0.209 Fixed ^b^ |
| Effect of age on CL (exponent) | -0.0145 (-0.0231–-0.00622) | - |
| Scaling factor for F (fold change) | - | 1.26 (1.09-1.47) ^g^ |
| Between-subject variability in CL (%) | 25.6 (20.6–32.5) | 25.6 Fixed ^b^ |
| Between-subject variability in Vc (%) | - | 23.5 Fixed ^b^ |
| Between-subject variability in Vp1 (%) | - | 29.6 Fixed ^b^ |
| Between-subject variability in Vp2 (%) | - | 54.6 Fixed ^b^ |
| Between-subject variability in F (%) | - | 30.1 Fixed ^b^ |
| Between occasion variability in F (%) | 17.3 (12.4–23.3) | 35.4 Fixed ^b^ |
| Between occasion variability in MTT (%) | 53.7 (35.8–70.8) | 46.6 Fixed ^b^ |
| Between occasion variability in Ka (%) | 95.3 (54.0–130) | 32.6 Fixed ^b^ |
| Proportional error for plasma (%) | 7.60 (6.66–8.81) | 11.4 Fixed ^b^ |
| Additive error for plasma (mg/L) | 0.136 (0.0255–0.249) | 1.56x10^-3^Fixed |
| CSF-to-plasma pseudo-partition coefficient, PPC_CSF-Plasma_  (.) | 0.690 (0.621–0.759) | 1.30x10⁻³ (1.13x10⁻³ – 1.47x10⁻³) |
| Plasma-to-CSF equilibrium half-life, t1/2_Plasma-CSF_ (h) | 4.65 (2.78–7.70) | 55.4 (22.3– 321) |
| Proportional error for CSF (%) | 25.5 (20.3–34.9) | 34.2 (26.4–49.1) |
| Additive error for CSF (mg/L) ^h^ | 4.00x10^-2^ Fixed | 1.00x10^-5^ Fixed |

*^a^Values in parentheses represent the 95% confidence interval, calculated using sampling importance resampling (SIR) on the final parameter estimates. This technique was not applied to the clofazimine plasma pharmacokinetic parameters used from the previous model by Abdelwahab et al*(3)*, as no estimation was performed in this analysis.*

*^b^Clofazimine plasma pharmacokinetic parameters were fixed at the values reported by the previously published model* (3)*.*

*^c^The parameter was allometrically scaled. The reported value refers to the typical individual in the cohort with fat-free mass of 37 kg.*

*^d^The parameter was allometrically scaled. The reported value corresponds to the typical individual in the cohort with a total body weight of 52 kg, which is equivalent to the value from the previous model for an individual with a total body weight of 54 kg.*

*^e^The parameter was allometrically scaled. The reported value corresponds to the typical individual in the cohort with a fat-free mass of 37 kg, which is equivalent to the value from the previous model for an individual with a fat-free mass of 40.3 kg.*

*^f^The parameter was allometrically scaled. The reported value corresponds to the typical individual in the cohort with a fat mass of 13 kg, which is equivalent to the value from the previous model for an individual with a fat mass of 11.2 kg.*

*^g^Scaling factor to account for differences in bioavailability between the two cohorts. The clofazimine plasma individual pharmacokinetic parameters are adjusted based on this new bioavailability value.*

*^h^The estimate of the additive component of the residual unexplained variability for the CSF observations did not significantly differ from its lower boundary of 20% of LLOQ, it was consequently fixed to this va*

**
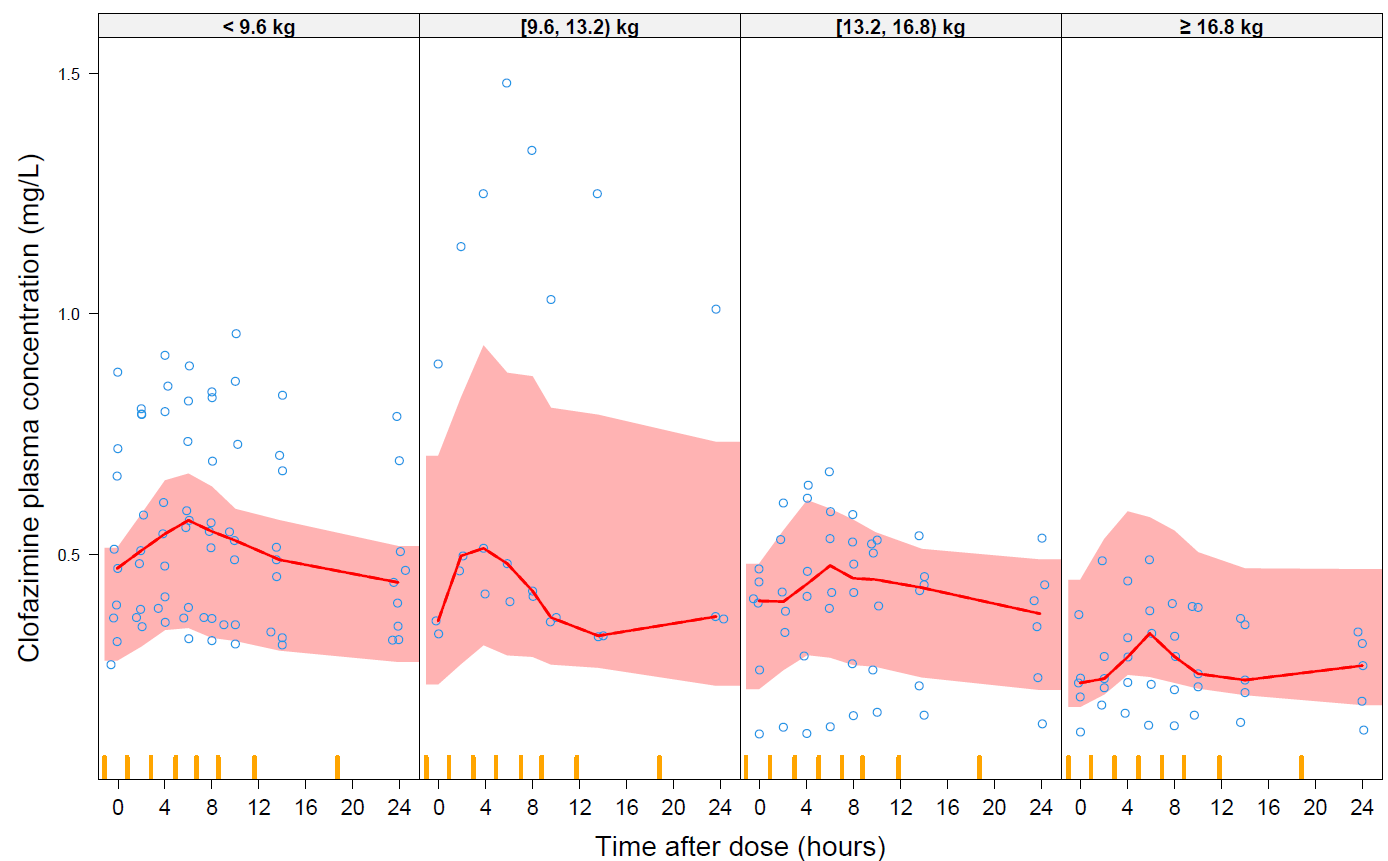
**

**Figure S6. Visual predictive check for clofazimine plasma concentrations, stratified by** fat mass, versus time after dose. The circles represent the observed data, the solid line represents the median of the observed data, and the shaded area represents the 95% confidence interval.


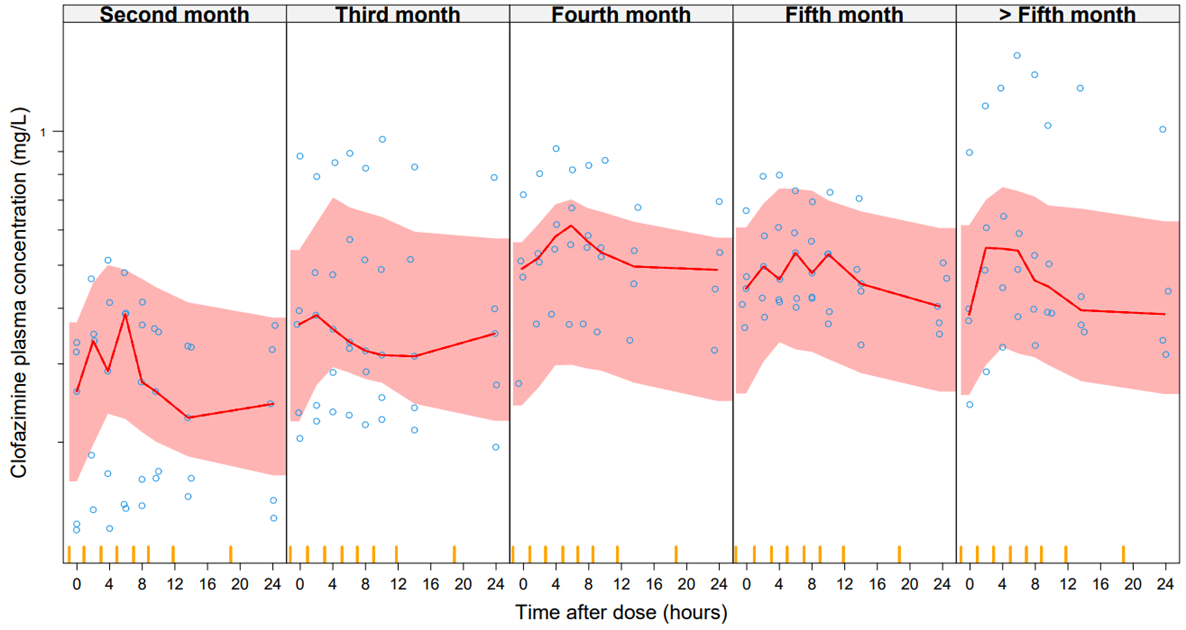


**Figure S7. Visual predictive check for clofazimine plasma concentrations, stratified by time on treatment, versus time after dose.** The circles represent the observed data, the solid line represents the median of the observed data, and the shaded area represents the 95% confidence interval

**
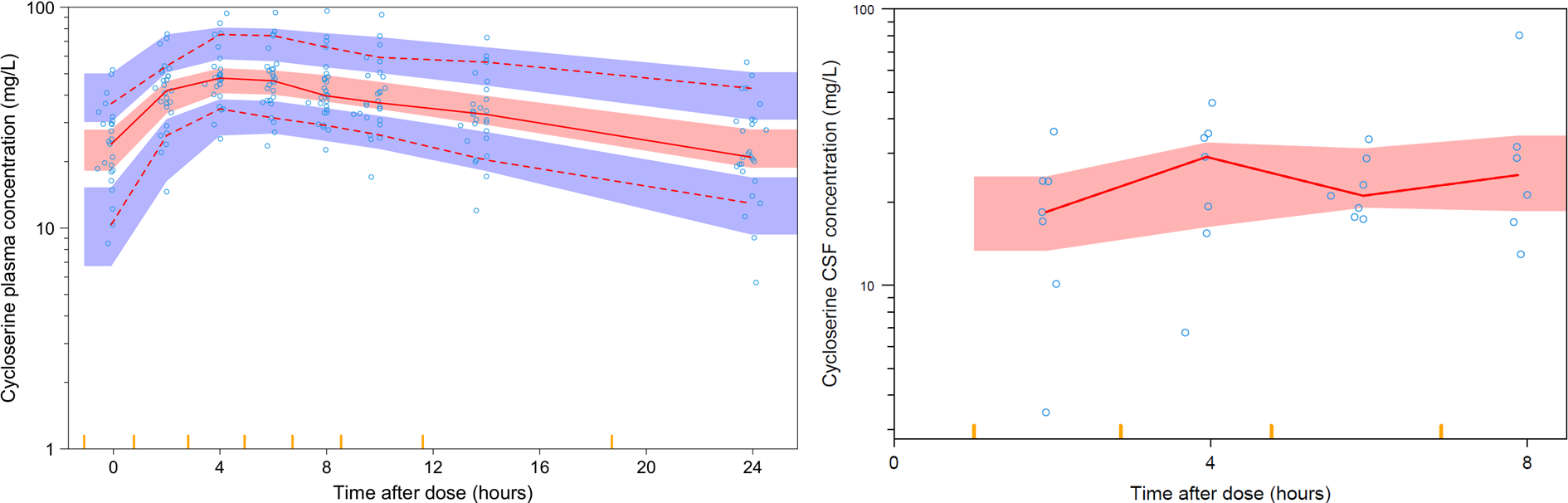
Figure S8. Visual predictive check for cycloserine plasma and cerebrospinal fluid (CSF) concentrations versus time after dose.** The circles represent the observed data, the dashed and solid lines are the 10^th^, 50^th^, and 90^th^ percentiles of the observed data, while the shaded areas represent the corresponding model-predicted 95% confidence intervals.

**Figure S9. Visual predictive check for clofazimine plasma and cerebrospinal fluid (CSF) concentrations versus time after dose.** The circles represent the observed data, the dashed and solid lines are the 10^th^, 50^th^, and 90^th^ percentiles of the observed data, while the shaded areas represent the corresponding model-predicted 95% confidence intervals.


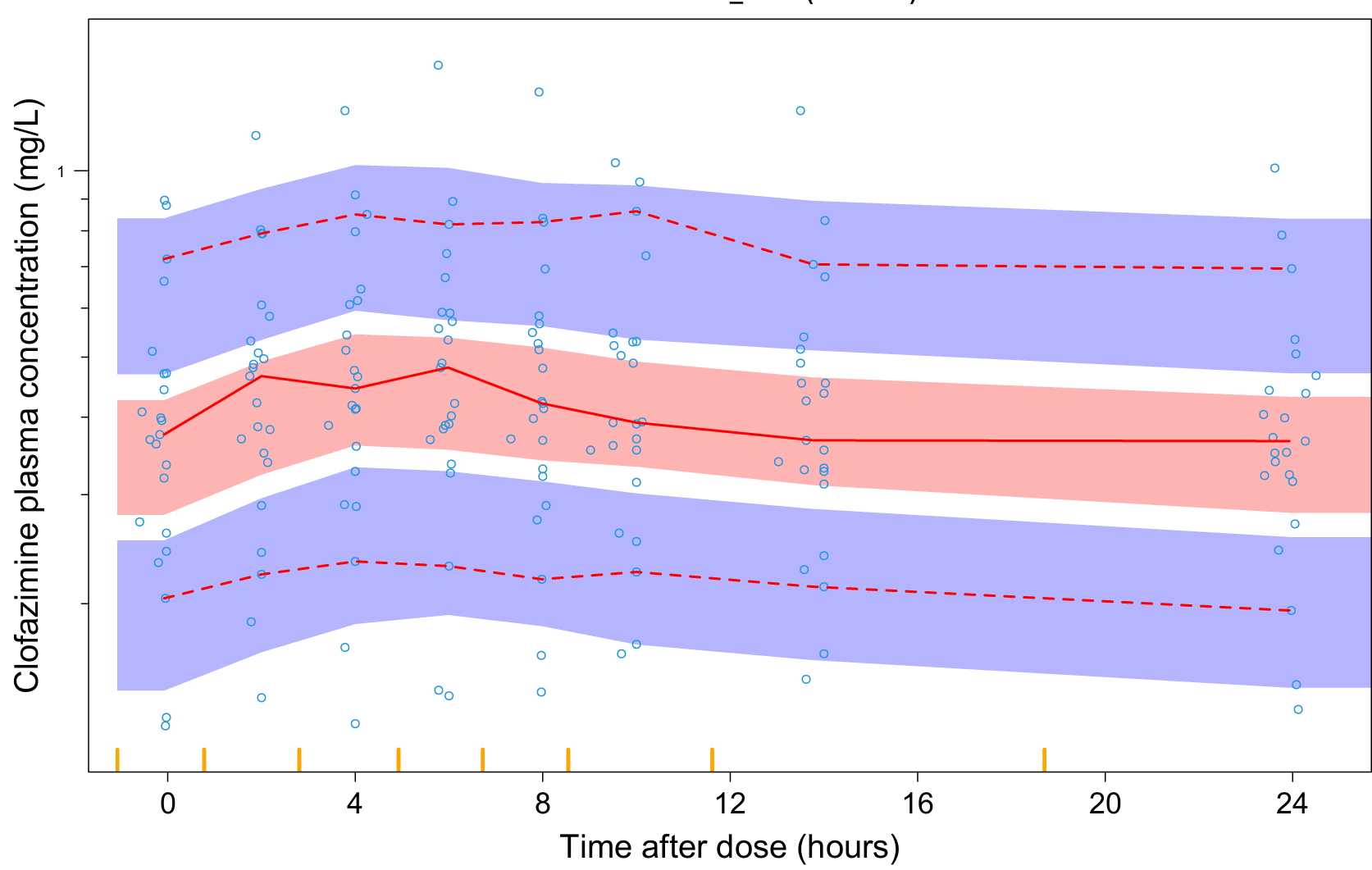

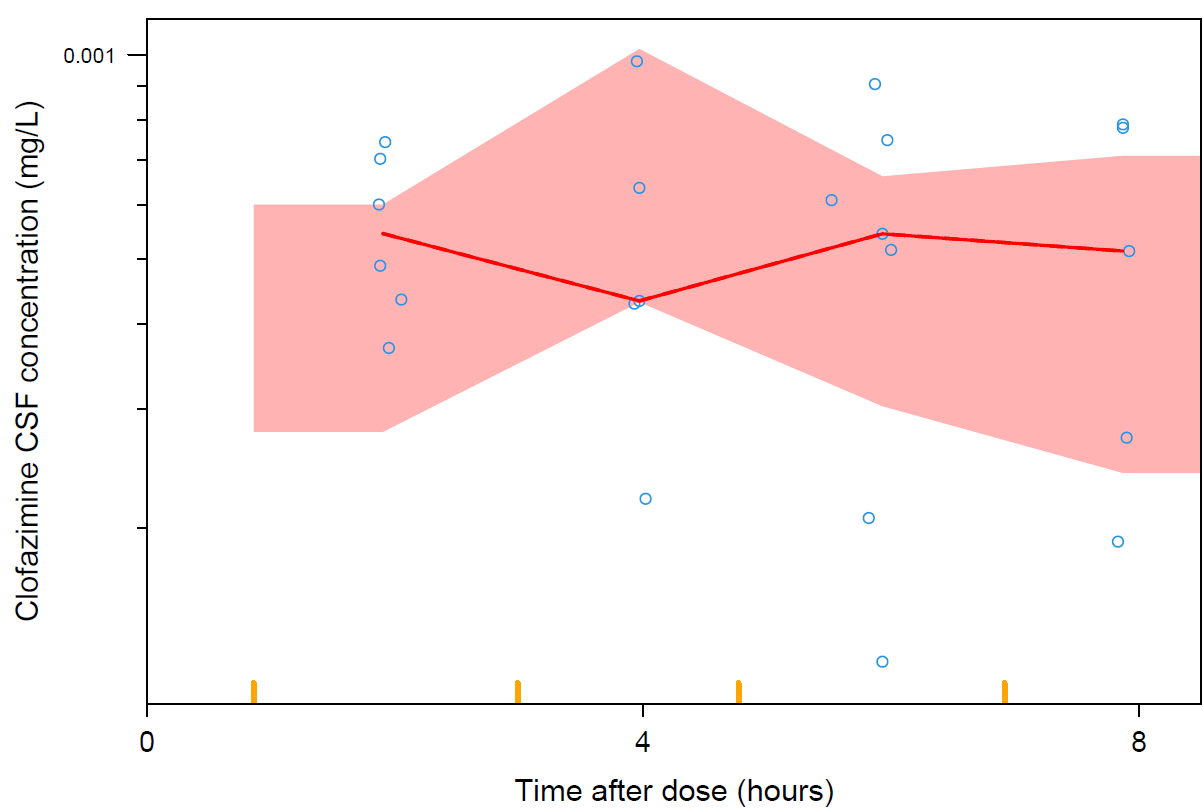


**
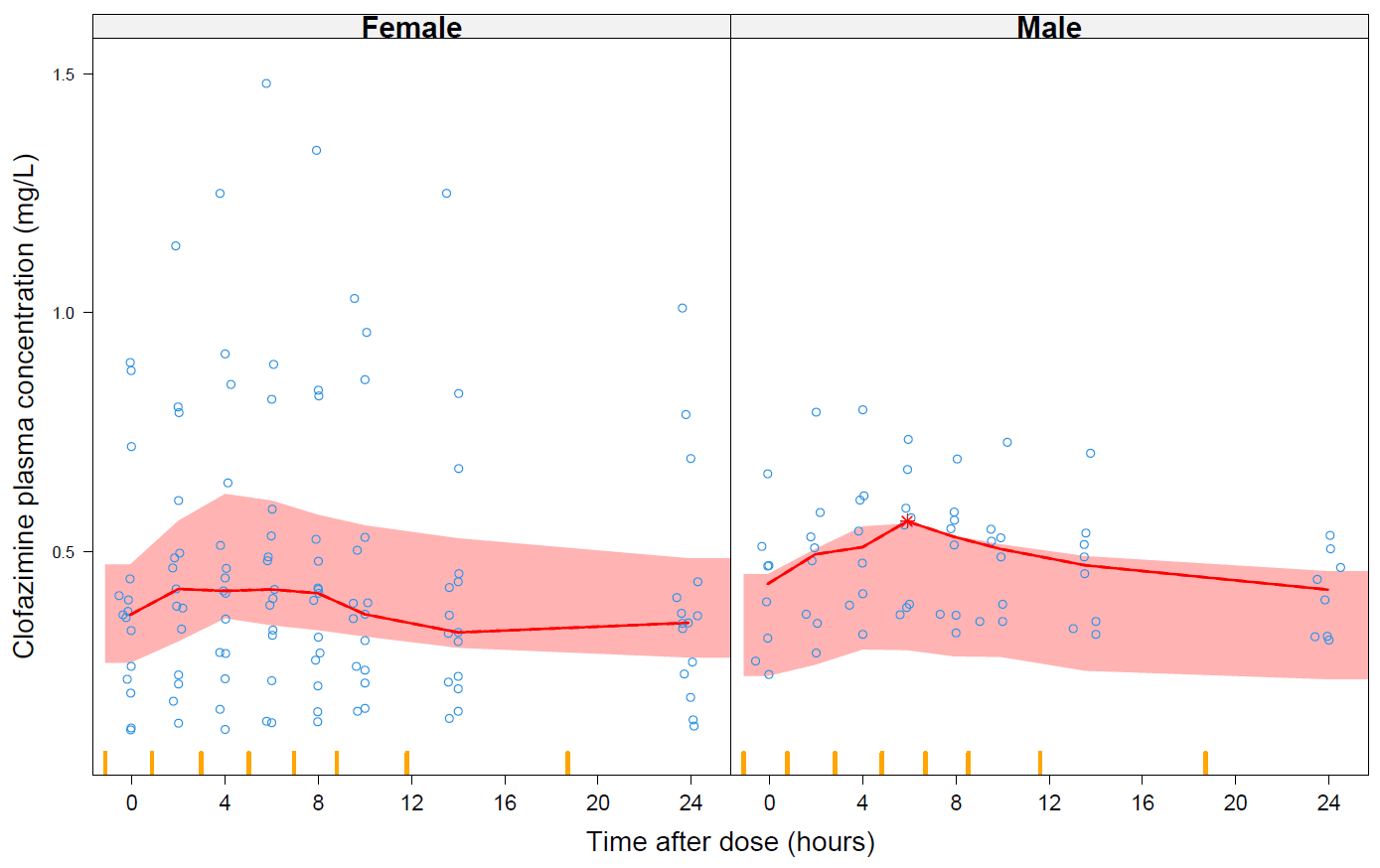
Figure S10. Visual predictive check for clofazimine plasma concentrations, stratified by sex, versus time after dose.** The circles represent the observed data, the solid line represents the median of the observed data, and the shaded area represents the 95% confidence interval.

**
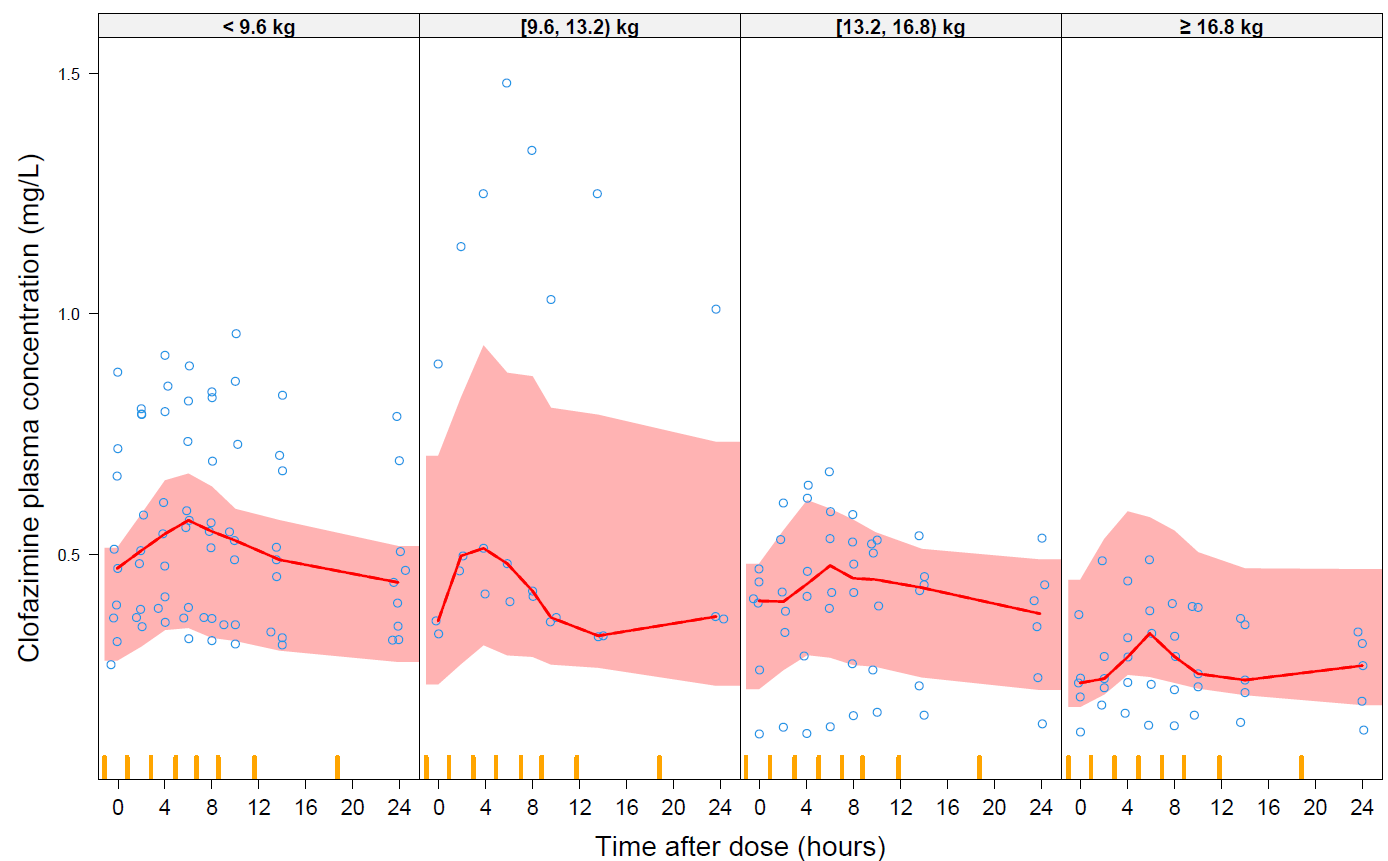
Figure S11. Visual predictive check for clofazimine plasma concentrations, stratified by fat mass, versus time after dose.** The circles represent the observed data, the solid line represents the median of the observed data, and the shaded area represents the 95% confidence interval.


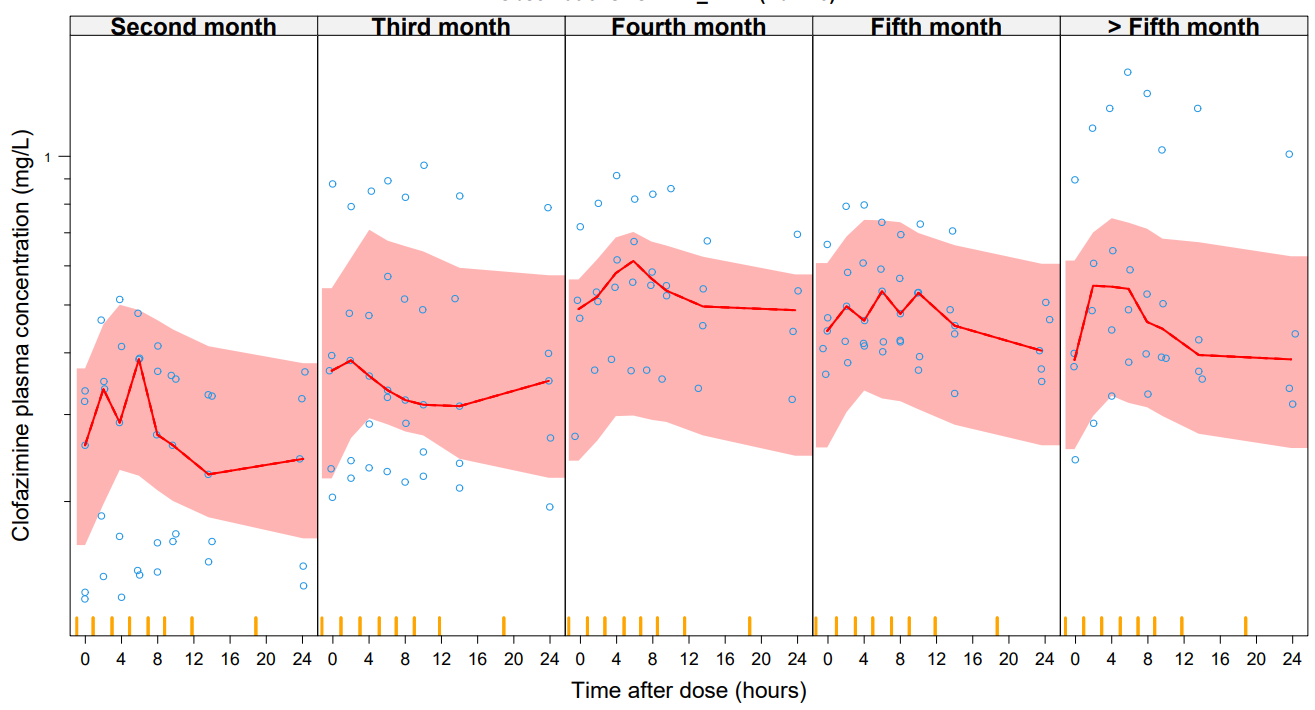


**Figure S12. Visual predictive check for clofazimine plasma concentrations, stratified by time on treatment, versus time after dose.** The circles represent the observed data, the solid line represents the median of the observed data, and the shaded area represents the 95% confidence interval.

**Effect compartment implementation for CSF concentrations modelling**

The CSF concentrations were modelled as dependent on the plasma concentrations, following the method suggested by Sheiner *et al*. (21). This modelling approach involved implementing a hypothetical effect compartment linked to the central compartment. This method assumes negligible drug transfer from the central to the effect compartment, as well as negligible volume of the effect compartment compared to the central compartment. The following differential equation summarizes the kinetic of the effect compartment:

$$\frac{{dC}_{CSF}}{dt}=k_{Plasma-CSF}\cdot\left( PPC\cdot C_{Plasma}-C_{CSF} \right)$$

The equilibrium between the central compartment (plasma) and the effect compartment (CSF) is governed by the first-order rate constant k_Plasma−CSF_. PPC represents the pseudo-partition coefficient, while C_Plasma_ and C_CSF_ denote the drug concentration in plasma and CSF at time 𝑡, respectively. The k_Plasma−CSF_ was used to calculate the plasma to CSF equilibration half-life (HL_Plasma-CSF_) employing the equation:

$${HL}_{Plasma-CSF}=\frac{ln\left( 2 \right)}{k_{Plasma-CSF}}$$

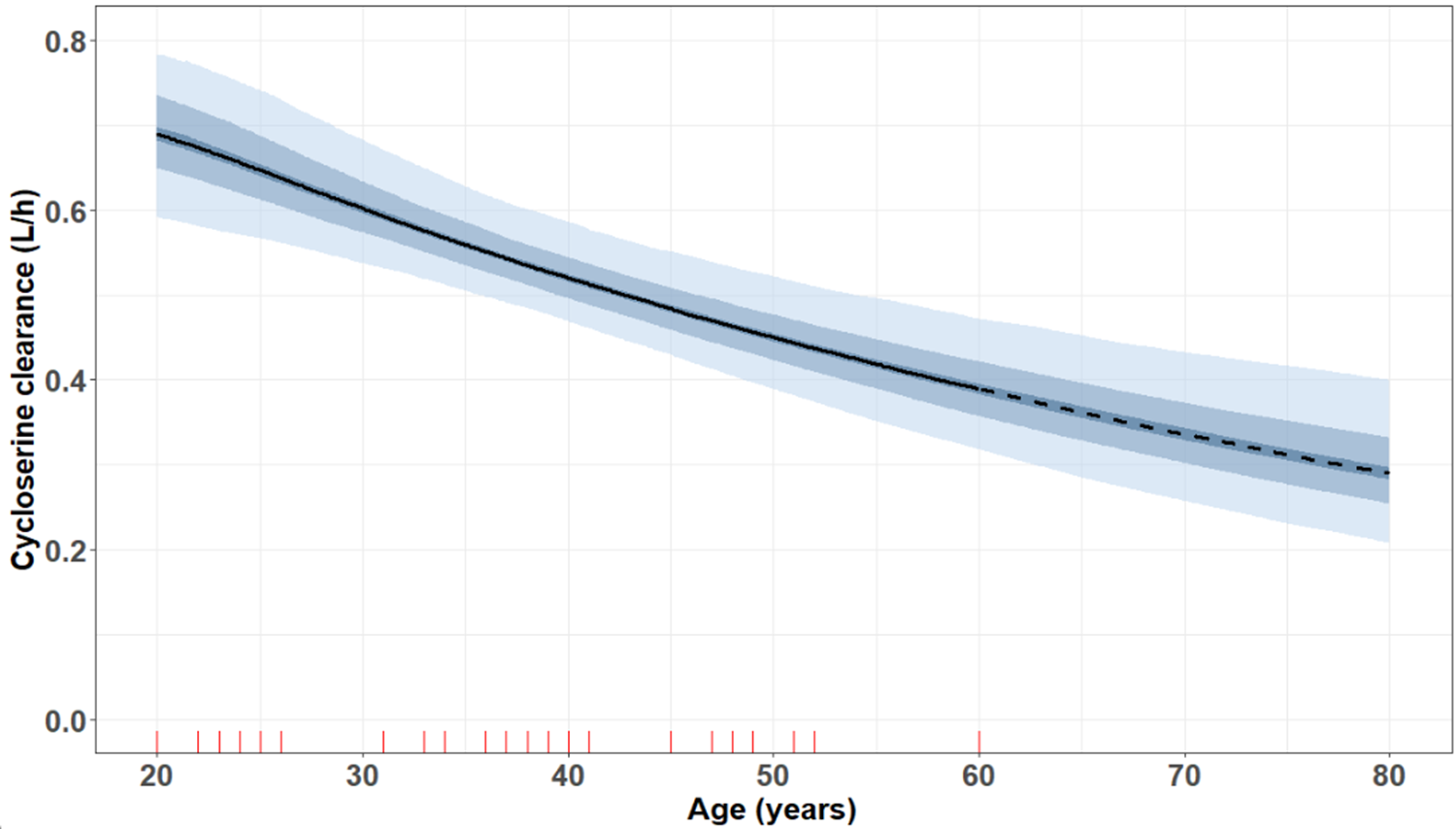


**Figure S13. Relationship between cycloserine clearance and age, modelled using an exponential decay function.**

The solid black line and red tick marks on the x-axis represent the observed age values in our cohort, while the dashed lines indicate extrapolations beyond the observed range. The shaded areas represent the 10th, 50th, and 90th prediction intervals of the predicted cycloserine clearance.

# S3: LC-MS/MS Assay methods and validation parameters

**Clofazimine**

**Plasma assay summary:** Clofazimine was analysed using a validated liquid chromatography-tandem mass spectrometry assay developed at the Division of Clinical Pharmacology, University of Cape Town.  The plasma assay consisted of a protein precipitation extraction, followed by high-performance liquid chromatography with tandem mass spectrometry detection.  The extraction procedure was followed by isocratic liquid chromatographic separation using a Phenomenex Synergi Hydro-RP (4 µm, 150 x 2.0 mm) analytical column.  A Sciex API 3000 mass spectrometer operating at unit resolution in multiple reaction monitoring mode was used to monitor the transition of the protonated precursor ions at m/z 472.9 and m/z 480.2 to the product ions at m/z 431.0 and m/z 432.1, respectively, for clofazimine and the internal standard.  The calibration curve was fitted with a quadratic regression (weighted by 1/concentration) over the range of 0.00781–2.00 µg/mL.  The accuracies (%Nom) ranged from 100.1% to 105.0%, with precision (%CV) less than 7.9% at the low, medium, and high-quality control concentrations during inter-batch validation.

**CSF assay summary:** Clofazimine was analysed using a validated liquid chromatography-tandem mass spectrometry assay developed at the Division of Clinical Pharmacology, University of Cape Town.  The cerebrospinal fluid assay utilized the addition of human plasma to samples to reverse nonspecific binding of clofazimine to polypropylene tubes. The extraction procedure consisted of a simple protein precipitation followed by high-performance liquid chromatography with tandem mass spectrometry detection. A gradient liquid chromatographic separation was achieved using a Restek Raptor biphenyl analytical column (2.1 mm x 50 mm, 2.7 µm).  A SCIEX 5500 triple quadrupole mass spectrometer operated in the positive mode at unit resolution enabled multiple reaction monitoring for the transitions m/z 473.0 to m/z 431.0 for clofazimine and m/z 480.0 to m/z 429.0 for the deuterated internal standard (clofazimine-d7). The calibration curve was fitted with a quadratic regression (weighted by 1/concentration) over the range of 0.0500–5.00 ng/mL. The accuracies (%Nom) ranged from 97.1% to 103.2%, with a precision (%CV) of less than 6% at the low, medium, and high-quality control concentrations for the inter-batch validation.

**Cycloserine: Quantitation of cycloserine in plasma and CSF**

Instrument: Derivatised cycloserine was quantified on a Shimadzu 8040 triple quadrupole mass spectrometer with a Shimadzu Prominence liquid chromatography system (Kyoto, Japan) and LabSolutions (Version 5.109) analysis software. Derivatised cycloserine and internal standard were detected at m/z 335.9/157.2 and z 340/158.15, respectively, in the positive ion mode.

Chromatography: Chromatographic separation was achieved on a Shim-Pack Velox Biphenyl column (2.1 x 100 mm, 2.7 μm; Shimadzu) housed at 30°C. Mobile phases consisted of (A) 2 mM ammonium formate and 0.1% formic acid in water and (B) 2 mM ammonium formate and 0.1% formic acid in acetonitrile:water (95:5) at a flow rate of 0.4 mL/min using a gradient method. The derivatised cycloserine eluted at ~3.3 min.

Plasma: Working solutions were prepared in water over a concentration range of 3.2 - 1200 µg/mL from an initial 2 mg/mL stock solution. A volume of 50 µL of each working solution was used to spike 950 µL of blank K_2_EDTA plasma to yield calibration standards ranging from 0.160 to 60.0 µg/mL. Similarly, working solutions were prepared and spiked into plasma to yield QC concentrations of 0.160 (LLOQ), 0.450 (QC low), 24.0 (QC medium) and 48.0 µg/mL (QC high). A volume of 20 µL of 10 µg/mL rac Cycloserine ^15^N d_3_ was added to 50 µL plasma, followed by the addition of 150 µL dansyl chloride at 2 mg/mL. The sample was vortexed for 30 s, centrifuged at 16 000 x g for 5 min at room temperature, after which 150 µL was transferred to a clean polypropylene tube. A volume of 100 µL of 100 mM ammonium bicarbonate (pH 9.5) was added and the sample was incubated at 50°C for 2 hours. A volume of 50 µL of 2% formic acid as added and the sample was cooled at 4°C for 15 min. The sample was vortex mixed for 10 s, and centrifuged at 16 000 x g for 5 min. A volume of 5 µL was injected onto LC-MS.

CSF: Working solutions were prepared in water over a concentration range of 4 - 1000 µg/mL from an initial 2 mg/mL stock solution. A volume of 50 µL of each working solution was used to spike 950 µL of blank surrogate CSF to yield calibration standards ranging from 0.20 to 50.0 µg/mL. Similarly, working solutions were prepared and spiked into surrogate CSF to yield QC concentrations of 0.200 (LLOQ), 0.400 (QC low), 20.0 (QC medium) and 40.0 µg/mL (QC high). For the extraction of cycloserine from CSF, 20 µL of 10 µg/mL rac Cycloserine ^15^N d_3_ was added to 50 µL of CSF. A volume of 150 µl of Dansyl chloride at 2 mg/mL was added to the sample, and vortex mided for 30 s, followed by centrifugation at 16 000 x g for 5 min. A volume of 150 µL of the supernatant was transferred to a microcentrifuge tube and 100 µL of 100 mM ammonium bicarbonate (pH 9.5) was added. The sample was heated at 50°C for 2 h, after which 50 µL of 2% formic acid was added, followed by cooling at 4°C for 5 min. The sample was centrifuged at 16 000 x g for 5 min and diluted with 5 mL 10 mM ammonium formate.  Solid phase extraction was performed with Waters Vacc 1cc (100 mg) tC18 cartridges, which were conditioned with 1 mL methanol and equilibrated with 1 mL 10 mM Ammonium formate. The derivatized sample was added to the SPE cartidges and washed with 1 mL water and 1 mL methanol:water (5:95, v/v) prior to elution with 1.5 mL methanol. The eluent was dried under nitrogen, reconstituted with 200 µL of water:acetonitrile (70:30, v/v), and 5 µL injected on LC-MS for analysis.
